# The Risk–Benefit Balance of Oral Corticosteroid Treatment for Asthma Attacks: A Discrete Choice Experiment of Patients and Healthcare Professionals in the UK and New Zealand

**DOI:** 10.1111/resp.70077

**Published:** 2025-06-30

**Authors:** Imran Howell, Jonathan Noble, Aleksandra Howell, Caitlin Morgan, Jennifer Logan, Sarah Miller, Rekha Chaudhuri, Richard E. K. Russell, Mona Bafadhel, Richard Beasley, Ian D. Pavord, John Buckell

**Affiliations:** ^1^ Respiratory Medicine Unit and Oxford Respiratory NIHR BRC, Nuffield Department of Medicine University of Oxford Oxford UK; ^2^ Medical Research Institute of New Zealand Wellington New Zealand; ^3^ North Bristol NHS Trust Bristol UK; ^4^ Gartnaval General Hospital Glasgow UK; ^5^ Patient and Public Representative Oxford UK; ^6^ King's Centre for Lung Health School of Immunology and Microbial Sciences, King's College London London UK; ^7^ Health Economics Research Centre, Nuffield Department of Population Health University of Oxford Oxford UK

**Keywords:** asthma, asthma attacks, asthma exacerbations, corticosteroids, glucocorticoids, patient preferences

## Abstract

**Background and Objective:**

Oral corticosteroids (OCS) are the guideline recommended treatment for all asthma attacks, but benefits must be considered alongside the potential for cumulative side‐effects. There is interest in trialling biomarker‐directed management of attacks to rationalise OCS treatment in those with least benefit. Understanding stakeholder perspectives on the risks and benefits associated with OCS treatment can inform trial design and shared decision‐making discussions in clinical practice. The aim was to examine patients' and healthcare professionals' preferences for the risks and benefits associated with OCS treatment for asthma attacks.

**Methods:**

Discrete choice experiment (DCE) by patients with asthma and HCPs in the UK and New Zealand. Preferences were analysed using logit models.

**Results:**

Eight hundred and twenty‐four patients and 171 HCPs completed the DCE. Avoiding the risks of permanent side effects had the greatest impact on treatment preference by patients and HCPs. Avoidance of side effects was weighted higher by patients than HCPs. Patients with uncontrolled asthma were more prepared to trade risk for benefit. Symptom recovery was the most valued clinical benefit to patients and HCPs. Patients preferred ‘improving lung function’ over ‘avoiding additional GP treatment or hospitalisation’, whereas HCPs preferred avoidance of further healthcare utilisation. Based on their responses we estimated the minimum clinically important difference for the treatment failure outcome at 20%.

**Conclusion:**

Patients and HCPs will trade‐off treatment benefits to avoid the side‐effects associated with OCS. The risk–benefit balance of OCS should feature in shared decision‐making discussions with patients experiencing outpatient asthma attacks. The findings support developing trials to personalise acute asthma treatment.

## Abstract 2

We used a discrete choice experiment to understand patients' and healthcare professionals' risk–benefit preferences for acute asthma treatment. Both groups were willing to trade off clinical benefits to avoid the side effects associated with OCS. The risk–benefit balance of OCS should feature in shared decision‐making discussions with patients experiencing outpatient asthma attacks.

**See related** editorial

## 1 Introduction

Asthma attacks diminish physical and mental health and cause a decline in lung function [1, 2]. For over 30 years, oral corticosteroids (OCS) have been the guideline‐recommended treatment for all asthma attacks. This is based on small, randomised, placebo‐controlled trials (RCT) in the emergency department (fewer than 500 patients total) demonstrating reduced relapse rates and accelerated symptom recovery [3]. However, OCS can cause temporary and cumulative side effects, which worry patients but are not always well recognised by healthcare professionals (HCPs) [4, 5, 6]. Moreover, OCS efficacy for attacks in primary care may have been overestimated because these trials were conducted in an era when only 20% of participants were treated with inhaled corticosteroids (ICS) [7], which have been shown to be an effective treatment in their own right [8].

Corticosteroids have the most clinical efficacy in patients displaying type‐2 airway inflammation [9, 10]. Type‐2 inflammation can be identified by raised blood eosinophils or fractional exhaled nitric oxide (FeNO) [11, 12, 13]. In COPD, prednisolone is not a clinically effective treatment for outpatient attacks associated with a low blood eosinophil count [14]. These findings raise the possibility of using a biomarker‐directed treatment approach to rationalise OCS use in outpatient asthma attacks where side‐effect harms may outweigh clinical benefits from treatment.

Understanding stakeholder perspectives on the risks and benefits associated with OCS treatment can inform clinical shared decision‐making discussions with patients, determine whether rationalising OCS use in acute asthma is an important goal, inform the choice of primary outcome(s) for trials of acute asthma treatment, and inform the target difference for non‐inferiority calculation [15]. To better understand these factors, we carried out a discrete choice experiment (DCE) involving patients with asthma and HCPs treating asthma as key stakeholders.

## 2 Methods

### 2.1 Background to Discrete Choice Experiment Methodology

Discrete choice experiments (DCEs) measure participants' preferences for treatment options by choosing between hypothetical treatment alternatives [16]. Treatment options in each choice are described by a set of characteristics (termed, ‘attributes’). Each attribute is described by a set of levels, such as different amounts of risk or benefit (Table 1). In this DCE, six attributes encompassed important side effects and clinical outcomes of OCS treatment.

**TABLE 1:** The descriptive system: Treatment benefit and side effect attributes with their respective levels.

Attribute	Levels
Chance you need more asthma treatment from your GP within 28 days	5%
	10%
	15%
Chance you need hospital treatment within 28 days	5%
	10%
	15%
Amount your asthma symptoms improve within 7 days	100%
	50%
	25%
Amount your peak flow (lung function) improves within 7 days	0%
	20%
	40%
Chance you get permanent side effects from treatment (weak bones, heart disease, and diabetes)	None
	High
Chance you get side effects while taking treatment (anxiety, insomnia, and indigestion)	None
	High

DCEs can measure preferences for treatment where no real‐world data exists, though based closely on existing treatments [17]. DCEs also correspond well with real‐world behaviours, yielding reliable clinical information [18].

### 2.2 Development of Choice Tasks

Experimental design followed best‐practice principles [19]. A systematic review of asthma attacks treatment outcomes and Cochrane reviews on the efficacy of OCS treatment informed a panel of potential treatment benefits [7, 20]. A rapid review of academic and grey literature identified the types and frequencies of side effects associated with OCS treatment for acute asthma [4].

Patient and public involvement was central to the design. A focus group of patients with asthma recruited through Asthma + Lung UK (*n* = 5) co‐produced the design by shaping the research questions, refining the choice and description of the attributes, and advising on the maximum number of attributes per choice task. One focus group member (SM) continued as a co‐author to help interpret the results from a patient perspective.

**FIGURE 1:**
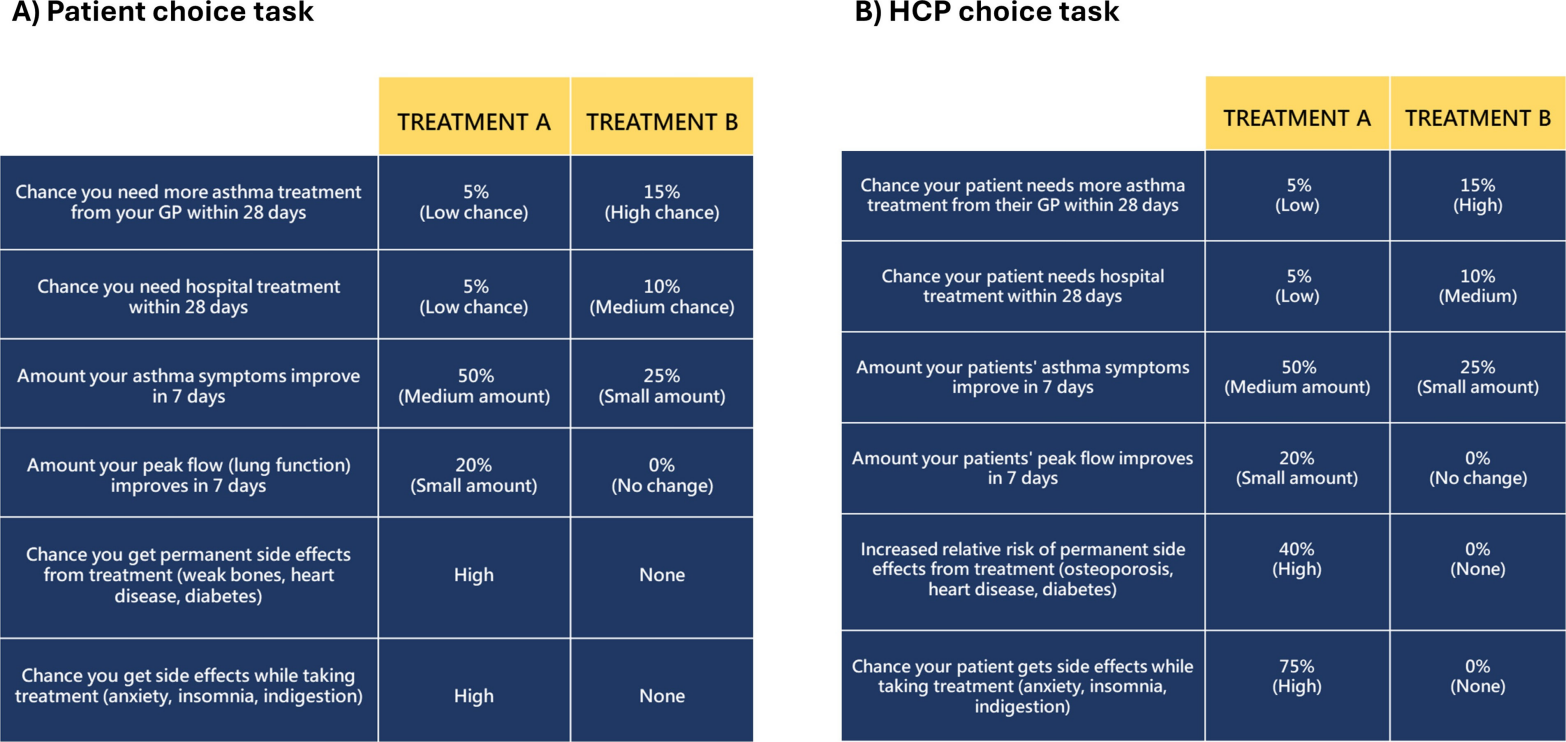
Example of the same choice task presented to both patients and HCPs.

Table 1 describes the attributes and levels in the design; Figure 1 shows an example of the choice tasks. The four treatment benefit attributes reflected important clinical outcomes to patients and HCPs. We chose quantitative levels to enable calculation of the risk–benefit balance. The levels for treatment failure to primary care and hospital were based on the incidence in randomised controlled trials and real‐world data [7, 21]. The levels for symptom improvement were based on randomised controlled trials [3]. The levels for peak expiratory flow (PEF) were extrapolated from the consensus agreement of minimum clinically important difference for FEV1 in short‐term trials (20%) and consensus guidelines specifying a > 20% short‐term variability of PEF as clinically meaningful in the diagnostic workup of asthma [3, 22, 23].

The two treatment side effect attributes were divided into the temporary and permanent side effects associated with OCS treatment, with three examples in each. This was based on feedback from the focus group where permanent side effects were considered different and more serious than temporary ones. We used binary, categorical classification (high/no risk) for patient participants to ease processing of risk information [24] and because the absolute risk of permanent side effects from one course of OCS is unknown and varies between individuals. With HCP respondents, we also included the estimated increased 10‐year relative risk of permanent side effects and the absolute risk of temporary side effects based on published literature [6, 25].

We used an unlabelled DCE (meaning that the alternatives had generic names of ‘treatment A’ and ‘treatment B’ rather than ‘OCS’ and ‘placebo’) because OCS are a guideline recommended treatment, which may bias against a placebo choice. Additionally, a placebo option should not have any side effects, which would limit the utility of the DCE for analysing the trade‐offs between benefits and risks.

Twenty‐four choice tasks were generated with a Bayesian D‐efficient design. Priors were obtained from a pilot study of 37 individuals. Participants were randomised to one of two blocks of 12 choice tasks because the focus group and pilot test participants felt that more than 12 tasks would cause cognitive fatigue, a recognised problem in DCEs [26]. All choice tasks faced by participants are listed in the supplement.

Respondents were given an introductory narrative with visual information to describe the tasks, attributes, and levels. To check if our explanation of the risks of asthma attacks, and risks and benefits associated with OCS influenced the results, we randomised a subgroup of patient participants into receiving two different wordings of introductory material with different emphasis on the risks and benefits. Explanation 1 had the benefits of OCS less emphasised, and Explanation 2 had the benefits of OCS more emphasised (including potentially reducing hospitalisation and death from asthma).

A practice choice task was presented prior to starting the real choice tasks. The DCE was distributed in an anonymous online survey (Qualtrics XM, London, UK) which also contained socio‐demographic questions and the Asthma Control Test (ACT) [27] in the patient sample. The wording of the patient task and introductory narrative sections had a reading age of 11 years (plain English) [28].

All participants were required to be aged 18 or above, and willing and able to give informed consent before participating (by reading a summary of the DCE and clicking ‘I agree’ on the first page). Participants were able to drop out at any point, and these responses were not included in the final data analysis. We used forced responses to prevent participants from skipping questions and inbuilt Qualtrics features to flag possible duplicate and ‘bot’ responses.

Ethical approval was granted for this study by the University of Oxford Central University Ethics Committee (Oxford, UK; Reference: R81047/RE001).

### 2.3 Sample Size

Using sample size calculations described by Bekker‐Grob [29] we prespecified a minimum sample of complete responses from 396 patients and 110 healthcare professionals.

### 2.4 Sampling

Participants who either had a self‐reported asthma diagnosis or were HCPs with self‐reported experience of treating asthma, were recruited in the United Kingdom and New Zealand.

Patients were recruited from UK asthma patient databases (Oxford, Glasgow, Bristol, London, Portsmouth, Southampton and Nottingham) and Wellington, New Zealand. We also recruited patients through advertisements by the Asthma + Lung UK charity and the Asthma and Respiratory Foundation of New Zealand.

We recruited UK HCPs by distributing the DCE to primary care staff through an advertisement by the Primary Care Respiratory Society, and the Association of Respiratory Nurses. We also distributed the DCE through professional networks of secondary care staff in regional respiratory departments, an Oxfordshire network of emergency department doctors, and respiratory and general medicine registrar training networks. We recruited New Zealand HCPs through professional networks at regional respiratory departments and an advertisement to GP networks.

### 2.5 Statistical Analysis

The full protocol and statistical analysis plan were published on the Open Science Framework prior to data collection [30]. Full methodological analysis is detailed in the supplement. Analyses were conducted in R (version 4.4.0) using the Apollo package (version 0.3.2).

We used multinomial logit (MNL) models as the primary analysis of experimental choices. MNL models assume that, for a given decision, people choose an alternative that maximises their utility (personal benefit). They calculate the mean utility and standard deviation in the sample for each attribute level.

Dummy‐coded attribute levels were independent variables (one level per attribute was set to zero as a reference against which the utility of the other levels were measured). A *t*‐ratio (two‐tailed Student *t*‐tests) < −1.96 or > 1.96 (equivalent to two‐sided *p* < 0.05) was statistically significant. Estimates were expressed as odds ratios by exponentiating MNL coefficients (logit models use the log of the odds ratio as a link function to calculate utility). The odds ratio indicates on average how likely participants were to choose a treatment that contained a specific level of risk or benefit, relative to its reference level (OR = 1), holding all else equal.

From the MNL models we tested interactions on pre‐specified socio‐demographic characteristics, marginal rates of substitution (MRS), and model forecasting.

MRS determine how much of a particular benefit individuals are willing to trade off in exchange for a reduction in a particular risk. We computed the MRS between treatment failure at 28 days (defined as the need for GP re‐treatment or hospitalisation within 28 days) against the risk of permanent side effects to inform the minimum clinically important difference (MCID) for this outcome [31]. The MRS represented the absolute increase in treatment failure that would be accepted in exchange for having no risk of OCS‐related side effects. The MRS was calculated from all HCPs and patients who had poorly controlled symptoms at the time of the survey (ACT < 15), representing those most likely to seek treatment.

Forecasting uses the estimated model to assess choices for hypothetical treatments [32]. We used the MNL model estimates for each attribute level to forecast choice probabilities of patients with poorly controlled asthma and HCPs choosing between a hypothetical OCS‐equivalent treatment versus a hypothetical placebo‐equivalent treatment. The estimated combination of levels for each treatment based on placebo‐controlled RCT data for OCS efficacy in acute asthma was as follows:

OCS‐equivalent: 5% relapse to GP, 5% relapse to hospital, 100% symptom improvement, 40% peak flow improvement, and high risk of permanent and temporary side effects.

Ineffective treatment (placebo‐equivalent): 15% relapse to GP, 15% relapse to hospital, 25% symptom improvement, 0% peak flow improvement, and no permanent or temporary side effects.

We used published data from two clinical trials to compare our model with observed real‐world behaviour of prednisolone prescribing for asthma attacks that were assessed by asthma specialists and assessed potential hypothetical bias [33, 34].

## 3 Results

Between April 2023 and December 2023, 1213 patient participants clicked on the survey link, 1149 consented to start the survey, and 824 patients completed the survey. Two hundred and seventy‐three HCPs clicked on the survey link, 239 consented to start the survey, and 171 completed the survey. A summary of patient and healthcare professional participant demographics are displayed in Table 2.

**TABLE 2:** Demographics for patient and HCP participants.

Characteristic	Patients (*n* = 824)	HCP (*n* = 171)
Mean age (range)	56 (18–86)	43 (27–70)
Gender		
Male	300 (36%)	66 (39%)
Female	518 (63%)	105 (61%)
Other	6 (1%)	0
Country		
UK	369 (45%)	116 (68%)
NZ	455 (55%)	55 (32%)
Ethnicity		NA
White	723 (88%)	
Non‐white	101 (12%)	
Mixed/multiple ethnic groups	44	
Asian	29	
Māori	12	
Pasifika	3	
Black/African/Caribbean	2	
Arab	1	
Hispanic	1	
Other	9	
Educational attainment		NA
School or vocational qualification	360 (44%)	
University degree or higher	464 (56%)	
Ever treatment with OCS for asthma		NA
Yes	610 (74%)	
No	214 (26%)	
Mean courses of OCS in last 12 months (SD)	1.3 (2.2)	NA
Ever hospitalised for asthma		NA
Yes	395 (48%)	
No	429 (52%)	
On biologic treatment for asthma		NA
Yes	185 (22%)	
No	639 (78%)	
Mean asthma control test score (SD)	18.4 (5.1)	NA
ACT > 15	592 (72%)	
ACT ≤ 15	232 (28%)	
Healthcare setting	NA	
Hospital		103 (60%)
General practice		48 (28%)
Emergency care		6 (4%)
Community		2 (1%)
Non‐clinical		12 (7%)
Healthcare role	NA	
Doctor (consultant)		50 (29%)
Doctor (General practitioner)		32 (19%)
Doctor (all training grades)		43 (25%)
Nurse		26 (15%)
Pharmacist		18 (11%)
Other		2 (1%)
Ever prescribed OCS for asthma	NA	
Yes		162 (95%)
No		9 (5%)

### 3.1 Multinomial Logit Model Results

Tables S1, S5, S6, S10, and S11 outline full model results for patients and HCPs.

**FIGURE 2:**
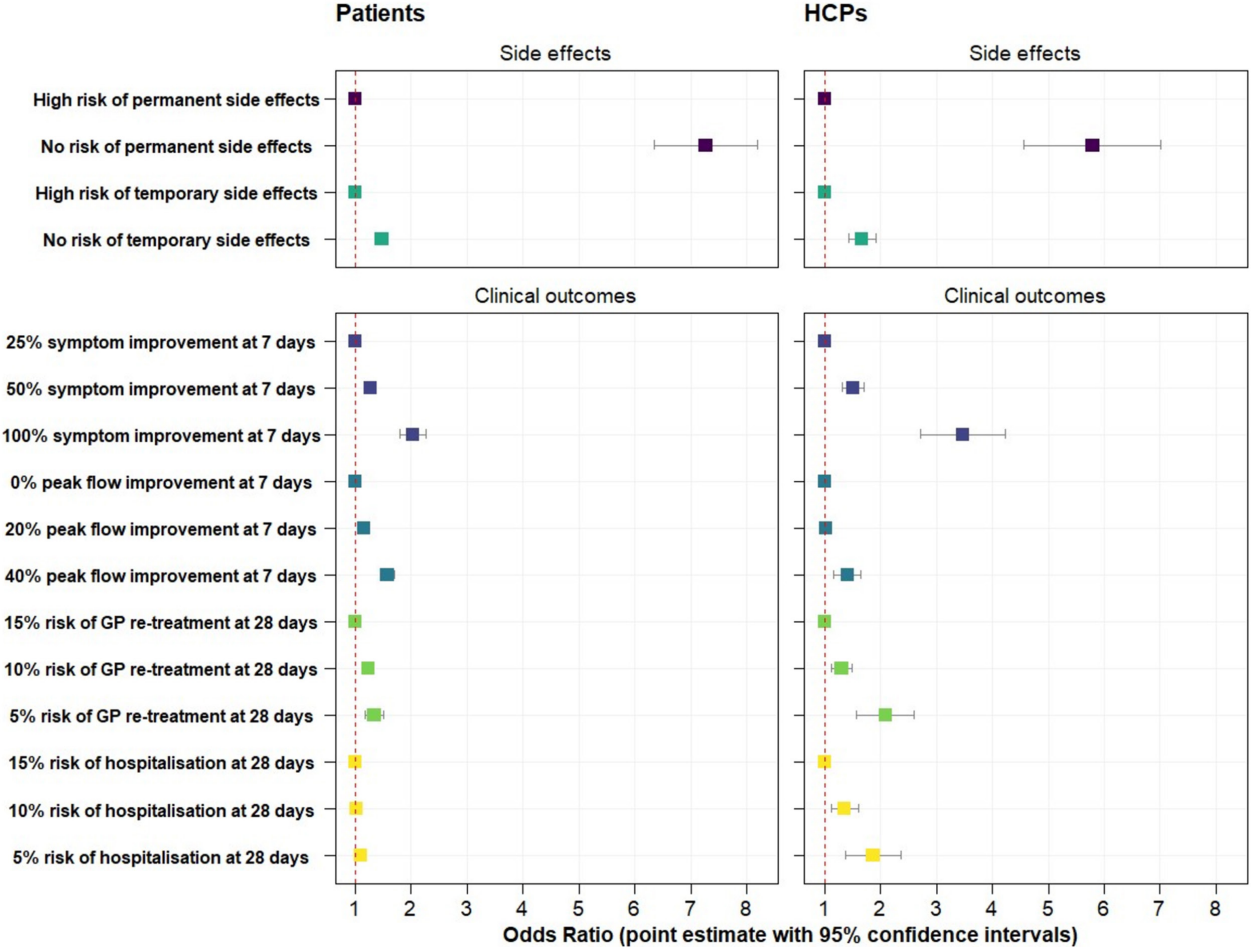
Comparing the preferences of patients and HCPs for different levels of risk and benefit from oral corticosteroid treatment for asthma attacks. The odds ratio indicates on average how likely participants were to choose a treatment that contained a specific level of risk or benefit, relative to its reference level (OR = 1), assuming all else equal. For example, patients were over seven times more likely to choose a treatment with no side effects risk compared to a treatment with a high risk of permanent side effects associated with OCS treatment.

Avoiding the risks of permanent side effects had the greatest impact on treatment preference by patients and HCPs (Table 3 and Figure 2). HCPs were willing to accept more side effect risk from treatment in exchange for more clinical benefits compared to patients.

**TABLE 3:** Preferences for different levels of risks and benefit.

		Patients			HCPs		
Attribute	Level	Odds ratio	95% Confidence intervals	*p*	Odds ratio	95% Confidence intervals	*p*
Risk of permanent side effects	None	7.27	6.35–8.20	< 0.001	5.79	4.57–7.02	< 0.001
	High (reference)	1.00	—	—	1.00	—	—
Risk of temporary side effects	None	1.48	1.36–1.59	< 0.001	1.67	1.42–1.91	< 0.001
	High (reference)	1.00	—	—	1.00	—	—
Symptom improvement at 7 days	100%	2.04	1.80–2.27	< 0.001	3.47	2.72–4.22	< 0.001
	50%	1.27	1.18–1.36	< 0.001	1.51	1.31–1.71	< 0.001
	25% (reference)	1.00	—	—	1.00	—	—
Peak flow improvement at 7 days	40%	1.57	1.45–1.70	< 0.001	1.41	1.16–1.65	< 0.001
	20%	1.16	1.07–1.24	< 0.001	1.02	0.87–1.18	0.386
	0% (reference)	1.00	—	—	1.00	—	—
Absolute risk of GP re‐treatment at 28 days	5%	1.34	1.17–1.50	< 0.001	2.09	1.57–2.60	< 0.001
	10%	1.24	1.16–1.31	< 0.001	1.30	1.12–1.48	< 0.001
	15% (reference)	1.00	—	—	1.00	—	—
Absolute risk of hospitalisation at 28 days	5%	1.10	0.96–1.23	0.075	1.86	1.37–2.36	< 0.001
	10%	1.02	0.93–1.10	0.363	1.36	1.12–1.60	0.002
	15% (reference)	1.00	—	—	1.00	—	—

Complete symptom recovery over 7 days was the most important clinical outcome to patients and HCPs. While patients and HCPs also preferred large (40%) improvements to their peak flow over 7 days compared to no improvement, these preferences were of less value than symptom recovery.

Patients slightly preferred treatment that reduced their absolute 28‐day risk of them needing to see their general practitioner for additional asthma treatment by 10%. However, patients did not factor a 10% absolute reduction in 28‐day risk of hospitalisation with acute asthma into their decision‐making. By contrast, HCPs strongly valued reducing healthcare utilisation in primary and secondary care.

### 3.2 Influence of Introductory Narrative

There was no significant difference in any of the results between the two groups randomised to receive different introductory narratives (Table S4). This implies that the way the benefits and risks of OCS treatment were presented to patients did not influence their treatment preferences.

### 3.3 Model Forecasting

The forecast result is displayed in Figure S1. Based on the MNL model results, patients with poorly controlled asthma (ACT < 15) were forecast to more frequently choose the hypothetical ineffective treatment with no risk of side effects (placebo‐equivalent) versus the hypothetical OCS‐equivalent treatment (predicted choice probability 57% vs. 43% respectively). By contrast, HCPs were forecast to more frequently choose OCS‐equivalent treatment versus placebo‐equivalent treatment (predicted choice probability 69% versus 31% respectively). The forecasted percentage of OCS usage by HCPs in our model aligned with real data from two trials where asthma attacks were assessed by asthma specialists and prednisolone was prescribed to approximately 60%–70% of patients [33, 34].

### 3.4 Marginal Rates of Substitution to Inform the Treatment Failure MCID for Future Trials

Patients with poorly controlled asthma were willing to accept a 58% (95% CI: 39%–78%) absolute increase in treatment failure in exchange for treatment without any permanent side effect risks associated with OCS (Tables S3 and S4).

HCPs were willing to accept a 26% (95% CI: 21%–32%) absolute increase in treatment failure within 28 days in exchange for treatment without any permanent side effect risks associated with OCS (Tables S8 and S9).

This suggests that the minimum important absolute treatment failure reduction with OCS acceptable to both patients and HCPs is approximately 20%. This can inform the non‐inferiority margin in future placebo‐controlled trials.

### 3.5 Interaction of Participant Socio‐Demographic Characteristics

**FIGURE 3:**
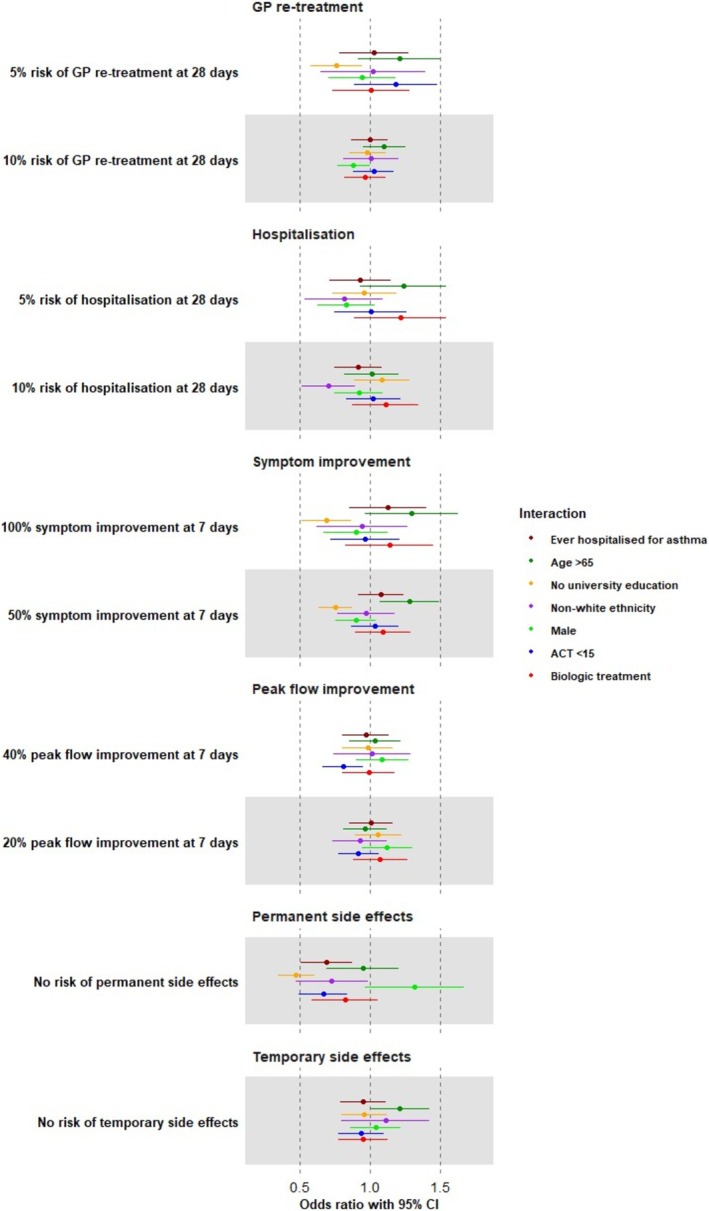
The interaction of socio‐demographic characteristics on patient preferences. Forest plot showing the influence of socio‐demographic factors on patient preferences for different levels of risks and benefits. An OR > 1 denotes stronger preference and an OR < 1 denotes weaker preference. For example, people who had previously been hospitalised for asthma had a lower OR (weaker preference) for a treatment with no risk of permanent side effects than people who had never been hospitalised for asthma.

Patients with poorly controlled asthma (OR vs. patients with well‐controlled asthma = 0.67, 95% CI 0.50–0.84), who had ever been hospitalised for asthma (OR vs. patients who have never been hospitalised = 0.69, 95% CI 0.51–0.87), and of non‐white ethnicity (OR vs. patients of white ethnicity = 0.73, 95% CI 0.48–0.98) put less value on treatments with no permanent side effects (Figure 3 and Table S2).

Patients aged over 65 years valued avoiding temporary side effects more strongly than those under 65 (OR vs. patients aged under 65 years = 1.22, 95% CI 1.01–1.42) and valued complete improvements in symptoms more strongly (OR vs. patients aged under 65 years = 1.30, 95% CI 0.97–1.63).

Patients who had not attended university generally made treatment choices that resulted in less favourable health outcomes. This included being less likely to choose treatment with complete symptom improvement (OR vs. patients who attended university = 0.69, 95% CI 0.52–0.86), less likely to choose treatment giving 10% absolute risk reduction of additional GP treatment (OR vs. patients who attended university = 0.76, 95% CI 0.58–0.94), and less likely to choose treatment with no permanent side effects (OR vs. patients who attended university = 0.48, 95% CI 0.35–0.61). We created a *post hoc* MNL model re‐weighted to reflect the proportion of university graduates in the UK/NZ population (30%). This revealed similar estimates to the original model and very similar risk–benefit balances (Table S12).

There was no difference in the treatment preferences of HCPs working in primary and secondary care settings (Figure S2 and Table S7). Male HCPs valued acute asthma treatment giving 10% absolute risk reduction in the 28‐day risk of hospitalisation (OR vs. female HCP = 2.52, 95% CI 1.16–3.87).

## 4 Discussion

### 4.1 Summary

Patients and HCPs value treatment for asthma attacks that avoids the side effects associated with OCS. In return, they are willing to trade off treatment benefits. HCPs were willing to accept more side effect risk from treatment in exchange for more clinical benefits compared to patients. Similarly, patients with poorly controlled asthma or a history of hospitalisation for asthma were willing to accept more risk of permanent side effects in exchange for treatment benefit compared to all other patients. While symptom recovery was the most important clinical outcome to HCPs and patients, HCPs preferred avoiding further healthcare utilisation more than patients.

### 4.2 Comparison to Prior Literature

We identified three relevant existing studies. A large UK survey identified that most patients experienced side effects from OCS and HCPs underestimated the frequency of these side effects [4]. A mixed‐methods study found that patients experienced extensive side effects from OCS that impacted their quality of life and led to psychosocial burden and social stigma [35]. A recent survey in France assessed patient perceptions of the risks and benefits of short course and maintenance OCS [36]. Although 40% of patients felt OCS to be efficacious, 80% experienced side effects. Most patients had a negative impression of OCS and were not satisfied with the side effect information provided by their doctor.

Our study builds on existing research by quantifying preferences for specific benefits and risks of treatment to understand the risk–benefit balance.

### 4.3 Implications

Our results inform current management of outpatient asthma attacks. Individual patients perceive the risks and benefits associated with OCS heterogeneously. This variability implies that some people may have a higher threshold before taking OCS than others. Variability of OCS use has also previously been recognised in a randomised trial of biomarker‐directed corticosteroid optimisation [37]. Interestingly, patients with lower educational attainment made treatment choices that resulted in less favourable health outcomes. This is consistent with findings from published literature and reinforces the importance of patient education when making treatment decisions in acute asthma [38, 39]. Potential treatment risks and benefits should be explained and weighed up with patients as decision‐making partners before initiating treatment. This education could take place when writing personalised action plans with patients.

Our findings also suggest that patients and HCPs would value further research into treatments and management that reduces acute OCS exposure. Prevention of asthma attacks with anti‐inflammatory treatment whilst reducing risk from lifestyle factors remains the primary goal. However, breakthrough attacks still occur, and the underlying pathology may not always be steroid‐responsive [40]. OCS treatment may have reduced clinical efficacy in outpatient attacks with low type‐2 inflammation and therefore an unfavourable risk–benefit balance to patients [9]. Biomarker‐directed, placebo‐controlled trials offer the opportunity to rationalise OCS treatment in those with least benefit, a concept already explored in COPD attacks [14]. Conversely, in breakthrough attacks with high type‐2 inflammation the rapidly acting and long‐duration anti‐IL‐5Rα monoclonal antibody benralizumab is an alternative acute anti‐inflammatory treatment to OCS [41]. Our DCE results suggests that evaluation of acute treatment leading to lower exposure to OCS side effects would be valued by both patients and HCPs.

Finally, the external validity of trials of acute asthma treatment is affected by the choice of clinical outcomes measured. Existing trials have tended to focus on physiological outcomes at short time‐points [20]. Our results demonstrated that symptom improvement over 7 days was the most valued clinical outcome of acute asthma treatment to both patients and HCPs. Interestingly, patients did not factor a 10% reduction of hospitalisation risk into their decision making. This may be because it is an uncommon event for most patients, so it was difficult to conceptualise, or because the 10% reduction was perceived as small. Subgroup analysis provides some support for these conclusions because both patients on biologics and those aged over 65—who were likely to have spent longer in hospital due to asthma—had near‐significant stronger preferences for a 10% hospitalisation risk reduction. Nonetheless, whilst healthcare utilisation outcomes were less important to patients, reducing the risk of GP re‐attendance or hospitalisation (treatment failure) was valued by HCPs, with a non‐inferiority margin of up to 20%. Together, the symptom and treatment failure outcomes may best reflect the combined preferences of patients and HCPs in future trials of asthma attack therapy.

### 4.4 Strengths and Limitations

Our study has several strengths. First, we obtained large samples from two countries with high asthma related morbidity and mortality, increasing the generalisability and relevance of our results [42, 43]. Second, by sampling both patients and HCPs, and considering their socio‐demographics, we could model differences in preferences towards the different treatment risks and benefits across groups, providing more clinically relevant information. Finally, patient involvement was a strong thread through the design, analysis, and interpretation of this research helping to ensure findings are important to patients.

Our study also has several limitations. First, using stated as opposed to revealed (i.e., real‐world) preferences during an asthma attack could introduce hypothetical bias in responses [32]. Integrating real preferences was not possible because clinical guidelines always recommend OCS treatment for an attack, which would bias prescription data. We mitigated this limitation by including the ACT at the time of completion. Despite patients with poorly controlled asthma tolerating more risk of permanent side effects, the overall message remained identical. Importantly, the forecasted percentage of OCS prescriptions issued by HCPs aligned with real prescription data from two trials where asthma attacks were assessed by asthma specialists, indicating the external validity of our findings [33, 34]. However, there may be less external validity of these findings in populations in other regions with different asthma treatment protocols or healthcare systems. Second, there may be unobserved factors that influenced choices. It is impossible to factor a participant's entire decision‐making process into a 6‐category choice‐task. We balanced the potential to over‐simplify the complexity of medical decision‐making against using too many attributes or levels which could confuse participants and impact data quality. This balance was informed by focus group feedback, literature review, and clinical expertise. Third, the small focus group size may have limited the ability to gather opinions from patients with different types of asthma or varying severity levels, limiting representativeness. Finally, using a categorical classification of side effects compared to quantitative levels for clinical benefits may introduce bias against treatment options with side effects. This was a pragmatic solution because the absolute risk of permanent side effects from one course of OCS is unknown and varies between individuals. To mitigate bias, each level of clinical benefit in the choice tasks also had a categorical classification (e.g., high, medium, and low chance).

In conclusion, our study demonstrates that patients and HCPs will trade off treatment benefits to avoid the side‐effects associated with OCS. In clinical practice, the risk–benefit balance of OCS should feature in shared decision‐making discussions with patients experiencing outpatient asthma attacks. The findings also support developing trials to personalise acute asthma treatment.

## Author Contributions

**Imran Howell:** conceptualization (lead), data curation (lead), formal analysis (lead), investigation (lead), methodology (lead), project administration (lead), visualization (lead), writing – original draft (lead), writing – review and editing (lead). **Jonathan Noble:** data curation (equal), investigation (equal), writing – review and editing (supporting). **Aleksandra Howell:** data curation (supporting), writing – review and editing (supporting). **Caitlin Morgan:** data curation (supporting), writing – review and editing (supporting). **Jennifer Logan:** data curation (supporting), writing – review and editing (supporting). **Sarah Miller:** conceptualization (supporting), writing – review and editing (supporting). **Rekha Chaudhuri:** data curation (supporting), writing – review and editing (supporting). **Richard E. K. Russell:** data curation (supporting), writing – review and editing (supporting). **Mona Bafadhel:** conceptualization (equal), investigation (equal), supervision (supporting), writing – review and editing (equal). **Richard Beasley:** data curation (equal), investigation (equal), writing – review and editing (equal). **Ian D. Pavord:** conceptualization (equal), funding acquisition (lead), investigation (equal), supervision (equal), writing – review and editing (equal). **John Buckell:** conceptualization (equal), formal analysis (equal), investigation (equal), methodology (equal), project administration (equal), supervision (lead), visualization (equal), writing – review and editing (equal).

## Ethics Statement

Ethical approval was granted for this study by the University of Oxford Central University Ethics Committee (Oxford, UK; Reference: R81047/RE001).

## Conflicts of Interest

I.H. has received a conference travel grant from GSK. He has also received grant funding from the BMA Foundation and is supported by the National Institute for Health and Care Research Oxford Biomedical Research Centre. A.H. has received a conference travel grant from GSK. She has also received grant funding from the National Institute for Health and Care Research. R.C. has received lecture fees from GSK, AZ, Teva, Chiesi, Sanofi, and Novartis; honoraria for Advisory Board Meetings from GSK, AZ, and Celltrion; sponsorship to attend international scientific meetings from Chiesi, Sanofi, and GSK; and a research grant to her Institute from AZ for a UK multi‐centre study. R.E.K.R. was supported by the National Institute for Health and Care Research Oxford Biomedical Research Centre and has received grant funding from Asthma+Lung UK and Circassia UK to his institution. He has received honoraria from AstraZeneca, GlaxoSmithKline, Sanofi, Chiesi, Zentiva, and Boehringer Ingelheim. He has received conference travel support from Chiesi and is a scientific advisor to AlbusHealth. M.B. has received grant funding from AstraZeneca, Asthma+Lung UK, Roche, GlaxoSmithKline, and the National Institute for Health and Care Research to her institution. She has received honoraria to her institution from AstraZeneca, Roche, and Sanofi, and has received conference travel support from AstraZeneca and Chiesi. M.B. is a scientific advisor to AlbusHealth and Areteia. R.B. has received institutional research funding from AstraZeneca, Cure Kids (NZ), Perpetual Guardian, Teva, and the Health Research Council of NZ, as well as personal fees from AstraZeneca, Avillion, Teva Pharmaceuticals, and Cipla outside the submitted work. R.B. is Chair of the Asthma Foundation of New Zealand adolescent and adult asthma guidelines group, a reviewer for the Global Initiative for Asthma, and was a member of the Board of Directors of the Global Initiative for Chronic Obstructive Lung Disease. R.B. is an Editorial Board member of Respirology and a co‐author of this article. He was excluded from all editorial decision‐making related to the acceptance of this article for publication. I.D.P. has received speaker's honoraria for speaking at sponsored meetings from Astra Zeneca, Boehringer Ingelheim, Aerocrine, Almirall, Novartis, Teva, Chiesi, Sanofi/Regeneron, Menarini, and GSK and payments for organising educational events from AZ, GSK, Sanofi/Regeneron, and Teva. He has received honoraria for attending advisory panels with Genentech, Sanofi/Regeneron, Astra Zeneca, Boehringer Ingelheim, GSK, Novartis, Teva, Merck, Circassia, Chiesi, and Knopp and payments to support FDA approval meetings from GSK. He has received sponsorship to attend international scientific meetings from Boehringer Ingelheim, GSK, Astra Zeneca, Teva, and Chiesi. He has received a grant from Chiesi to support a phase 2 clinical trial in Oxford. He is co‐patent holder of the rights to the Leicester Cough Questionnaire and has received payments for its use in clinical trials from Merck, Bayer, and Insmed. In 2014–5 and 2019–20, he was an expert witness for a patent dispute involving Astra Zeneca and Teva. The authors declare no conflicts of interest.

## Supporting information


**Data S1.** Supporting Information.

## Data Availability

Data and code scripts are available on request.
