# Supplementary material for: The Risk–Benefit Balance of Oral Corticosteroid Treatment for Asthma Attacks: A Discrete Choice Experiment of Patients and Healthcare Professionals in the UK and New Zealand
Source: Respirology. 2025 Jun 30;30(11):1035–45. doi: 10.1111/resp.70077 (PMC12581093; doi:10.1111/resp.70077)
Supplement: Supplementary file 1 — Data S1. Supporting Information. [file RESP-30-1035-s001.docx]

# Supplementary information

**The risk-benefit balance of oral corticosteroid treatment for asthma attacks: A discrete choice experiment of patients and healthcare professionals in the UK and New Zealand**

Imran Howell^1^, Jonathan Noble^2^, Aleksandra Howell^1^, Caitlin Morgan^3^, Jennifer Logan^4^, Sarah Miller^5^, Rekha Chaudhuri^4^, Richard E. K. Russell^6^, Mona Bafadhel^6^, Richard Beasley^2^, Ian D Pavord^1^, John Buckell^7^

**Affiliations**

1. Respiratory Medicine Unit and Oxford Respiratory NIHR BRC, Nuffield Department of Medicine, University of Oxford

2. Medical Research Institute of New Zealand, Wellington, New Zealand

3. North Bristol NHS Trust, Bristol, UK

4. Gartnaval General Hospital, Glasgow, UK

5. Patient and Public representative, Oxford, UK

6. King’s Centre for Lung Health, School of Immunology and Microbial Sciences, King’s College London, UK

7. Health Economics Research Centre, Nuffield Department of Population Health, University of Oxford

**Corresponding author**

Dr Imran Howell

Email: Imran.howell@ndm.ox.ac.uk

## Supplementary methods

### Multinomial logit model

In a DCE, the observed data from choices are in discrete, categorical form for each individual over a discrete set of alternatives. Therefore, linear regression is not appropriate for analysis because it relies on continuous data. To resolve this issue, the dichotomous dependent variables must be transformed into continuous variables (link function)^1^. Logit models use the log of the odds ratio as a link function to create latent variables. The logit model predicts the probability of an outcome occurring. Where the categorical dependent variable relates to choices, the latent variable is termed utility^1^.

In economics, there are broadly two types of utility – cardinal and ordinal. Cardinal utility asserts that the utility obtained from an alternative is measurable and the magnitude of the measurement is meaningful. For example, if a person gained 10 utils from eating an apple but 5 utils from eating an orange, then the person gets precisely half the pleasure from eating an orange^1^. As an example, cardinal utility is used in the calculation of quality adjusted life years (QALYs). Ordinal utility asserts that the utilities obtained from two alternatives indicate the order and intensity of preference, but the values themselves are meaningless. Using the example above, the conclusion is that the person prefers apples, but it is not possible to determine exactly by how much. This is termed relative utility and forms the basis of discrete choice models, such as MNL models^1^.

Random utility theory states that, for a given decision, people attempt to make rational choices between different options that maximise their utility (personal benefit)^1^. Stated choice modelling uses a series of trade-offs under experimental conditions to estimate the values of these utilities for different aspects of a choice. In this experiment, the two unlabelled choices were described by four clinical benefit attributes of OCS treatment in acute asthma, and two risks – temporary and permanent side effects of OCS treatment.

The utility that a respondent *i* derives from choosing alternative *j* in choice scenario *s* can be calculated as:

U*_ni =_ V_ni +_* 𝜀*_ni_*

In this equation, V*_ni_* represents the predictable (deterministic) part of the utility and 𝜀*_ni_* represents random, unobservable characteristics that cause deviation from modelled utility for alternative i and respondent n.

*V_ni_* = f (x_n,I_, z_n_, β)

Where x_n,I_ are characteristics of alternative i, as faced by decision maker n, z_n_ are characteristics of decision maker n, and β is a vector of estimated parameters.

In our experiment, *V* can be calculated as:

ASC_left-to-right-bias_ + β_risk of GP re-treatment_ * risk of GP re-treatment + β_risk of hospitalisation_* risk of hospitalisation + β_symptom improvement_ * symptom improvement + β_peak flow improvement_ * peak flow improvement + β_risk of temporary side effects_* risk of temporary side effects + β_risk of permanent side effects_* risk of permanent side effects

In the above utility function, ASC_left-to-right-bias_ denotes the alternative specific constant that captures the respondent’s tendency to choose alternatives presented on the left rather than the right. β are attribute-specific preference parameters. Each attribute was dummy coded such that one level per attribute was set to zero as a reference against which the utility of the other levels were measured. For example, the attribute symptom improvement had three levels: 25% improvement, 50% improvement, and 100% improvement in 7 days. The 25% improvement level was set to zero and the 50% improvement and 100% improvement parameters were estimated relative to 25% improvement.

The MNL model makes several assumptions^1^. First, we assume that the decision maker chooses an alternative *j* if it delivers the highest utility compared to the other alternative in our choice set. Second, the random error component (𝜀*_isj_*) is assumed to be independently (no correlation across alternatives) and identically (across alternatives and respondents) distributed using Type I extreme value distribution (Gumbel distribution). Third, the MNL model follows the Independence of Irrelevant Alternatives (IIA) theory which states that the choice between alternative A and alternative B should not depend on the quality of a third, unrelated alternative.

The MNL takes the following form:

$$P_{n,i}=\frac{e^{\mu V_{n,i}}}{\sum_{j=1}^{J} e^{\mu V_{n,j}}}$$

Where *P_n,i_* is the probability that decision maker n choosing alternative i from choice task *J*.

### Preference variation

Preference variation for the different risk and benefit attributes was modelled deterministically. Each attribute level was interacted with individual characteristics listed in our pre-specified analysis plan that were thought to potentially influence decision making. For patients this included: Ever being hospitalised for asthma, age (continuous and categorical covariates), educational attainment, ethnicity, gender, ACT score at the time of the DCE (continuous and categorical covariates), current treatment with monoclonal antibody (biologic) drugs. For HCPs this included: Age (continuous and categorical covariates), gender, type of HCP, workplace setting. Models were specified with all the listed interactions. The parameters measured in the MNL indicate attribute preference variation according to the specified individual characteristics.

### Marginal rates of substitution

Because decision makers are assumed to choose the alternative which maximises their utility or minimises their disutility, they are also assumed to trade between attributes. Bad performance of one attribute can be compensated by good performance of another attribute. Marginal rates of substitution (MRS) determine what change in one attribute is required to counteract a change in another while keeping utility the same^2^. Marginal utilities were given by a linear calculation of partial derivatives of utility, using the Delta method to estimate the standard errors:

$$\frac{\beta_{risk of treatment failure}}{\beta_{risk of permanent side effects}}=MRS$$

MRS were determined for treatment failure benefit versus the risk of permanent side effects from OCS to inform the minimum clinically important difference (MCID), and therefore non-inferiority margin, for future trials of OCS for asthma attacks^3^. Treatment failure was defined as combined GP re-treatment and hospitalisation similar to previous definitions in randomised controlled trials and modelled as a continuous covariate^4-6^. Since the permanent side effects attribute was categorical, the MRS represented the absolute increase in treatment failure that would be accepted in exchange for having no risk of OCS-related side effects. MRS for the symptom and peak flow parameters were not estimated because the MCID for commonly used asthma symptom questionnaires and measures of lung function are already established^7^.

### Testing scale between different samples

The scale of a model is inversely proportional to variance of error term (𝜀_ni_). A higher scale means there is a more deterministic choice process. The scale parameters will differ if more than one data set is compared which may confound the parameter estimates^8^. In these cases, it is important to rule out, or account for, difference in scale prior to concluding that the observed parameter differences are real. Identifying the difference in scale between datasets is achieved by normalising the variance in one group and identifying the relative scale parameter of the other group^9^.

In our data, we tested the scale factor between the subgroup randomised Explanation 1 and Explanation 2, between the UK and NZ samples in both the patient and HCP groups, and between the entire patient and HCP samples.

### Forecasting

Forecasting uses the estimated model to look at choices for hypothetical settings^10^. Predicted probabilities use the model estimates and specific attribute values to forecast for each treatment. We used the fitted MNL model to forecast choice probabilities of patients and HCPs choosing different treatments asthma attack treatment: OCS and no treatment (placebo). The estimated combination of levels for each treatment were as follows:

OCS: 5% relapse to GP, 5% relapse to hospital, 100% symptom improvement, 40% peak flow improvement, and high risk of permanent and temporary side effects.

No treatment (placebo): 15% relapse to GP, 15% relapse to hospital, 25% symptom improvement, 0% peak flow improvement, and no permanent or temporary side effects.

We used published data from two clinical trials to compare our model with observed real-world behaviour of prednisolone prescription for asthma attacks that were assessed by asthma specialists^11 12^. We know from these studies that the prednisolone prescription rate was approximately 60-70%. We used this estimate to assess potential hypothetical bias in the HCP MNL model.

### Sensitivity analysis with mixed logit models

The MNL model has advantages in that it is more straightforward to conceptualise. However, because it assumes that the deterministic part of utility remains constant across individuals (point estimate from sample), it is unable to account for heterogeneity in preferences between individuals (taste heterogeneity). Not accounting for heterogeneity can increase the error in the model and induce bias^13^. Taste heterogeneity can be subcategorised as deterministic or random. As stated above, deterministic heterogeneity was assessed with interactions based on individual characteristics. Random preference heterogeneity was modelled using a continuous mixing distribution (mixed logit model)^14^. The mixed logit allows relaxation of the IIA assumption. This is useful for stated preference data because there is a potential over the 12 choice sets that there are correlated responses that may violate IIA (for example, learning and inertia effects)^13^. In the mixed logit, parameters were treated as being normally distributed, with a mean and standard deviation estimated for each attribute. 2000 draws were taken to simulate the distribution of the parameters using the Modified Latin Hypercube Sampling algorithm^15^.

### Introductory narratives for patients and HCPs

#### Patient Explanation 1 (benefits of prednisolone less emphasised)

**About treatment for asthma attacks**

Steroid tablets are the main treatment for asthma attacks.

**Benefits of tablet steroids**

- They reduce the chance you need more asthma treatment within 1 month from your GP or at hospital from 15% (1 in 6 people) to 10% (1 in 10 people)
- They reduce the time taken for asthma symptoms to go away from 10 days to 7 days
- Lung function is sometimes measured during an asthma attack with a peak flow meter (see picture below). Steroids sometimes improve lung function.

**Risks of tablet steroids**

The risk of side effects from steroids tablets is high.

- 3 out of 4 asthma patients experience temporary side effects during steroid tablet treatment such as anxiety, difficulty sleeping, and indigestion
- Each course of steroid tablets makes it more likely that you will develop permanent side effects. 3 courses of steroid tablets nearly double the chance you will get heart disease, diabetes, and weak bones in your lifetime.

*<Picture of a person using a peak flow meter>*

**Instructions**

You will now be asked to choose between 12 sets of imaginary treatments for asthma attacks. These treatments are made up from 4 important outcomes and 2 risks. You will need to choose the option between Treatment A or Treatment B. Please choose the option you prefer the most, even if both options seem similar.

You will first see a test question to show you how the choices are presented.

If you are using a mobile device, please turn the device on its side.

#### Patient Explanation 2 (benefits of prednisolone more emphasised)

**About treatment for asthma attacks**

There is a risk of hospitalisation and death from asthma attacks. Steroid tablets are the main treatment for an asthma attack.

**Benefits of tablet steroids**

- They reduce the chance of being admitted to hospital for an asthma attack by around 50%
- They reduce the chance you need more asthma treatment within 1 month of an asthma attack
- They reduce the time taken for asthma symptoms to go away from 10 days to 7 days
- They sometimes improve lung function after an asthma attack. This can be measured with a peak flow meter (see picture below)

**Risks**

The risk of side effects from steroids tablets is high.

- 3 out of 4 asthma patients experience temporary side effects during steroid tablet treatment such as anxiety, difficulty sleeping, and indigestion
- Steroid tablets are also associated with long-term side effects. These include diabetes, heart disease, and weak bones. The risk of developing a long-term side effect increases with each course of steroid tablets

*<Picture of a person using a peak flow meter>*

**Instructions**

You will now be asked to choose between 12 sets of imaginary treatments for asthma attacks. These treatments are made up from 4 important outcomes and 2 risks. You will need to choose the option between Treatment A or Treatment B. Please choose the option you prefer the most, even if both options seem similar.

You will first see a test question to show you how the choices are presented.

If you are using a mobile device, please turn the device on its side.

#### HCP explanation

**About treatment for asthma attacks**

Oral steroids are the main treatment for an asthma attack.

**Benefits**

Placebo-controlled randomised controlled trials show that oral steroids:

- Reduce relapse (an unplanned visit for more asthma treatment to their GP or hospital) within 1 month from about 15% (1 in 6) to 10% (1 in 10)^1^
- They reduce the time taken for asthma symptoms improve from 10 days to 7 days^2^
- There is no significant improvement to lung function at 7 days^1^

**Risks**

The risk of side effects from steroids tablets is high.

- 75% of UK asthma patients experience at least one temporary side effects during steroid tablet treatment such as anxiety, difficulty sleeping, and indigestion^3^
- After a course of oral steroids, the 10-year relative risk of heart disease, diabetes, and osteoporosis is approximately 40% higher^4^

**Instructions**

You will now be asked to choose between 12 sets of imaginary treatments for asthma attacks. These treatments are made up from 4 important outcomes and 2 risks. Please choose whether you would prefer to prescribe either Treatment A or Treatment B, even if both options seem similar.

You will first see a test question to show you how the choices are presented.

If you are using a mobile device, please turn the device on its side.

**References**

1. Rowe BH, Spooner C, Ducharme F, et al. Corticosteroids for preventing relapse following acute exacerbations of asthma. Cochrane Database of Systematic Reviews 2007(3) doi: 10.1002/14651858.CD000195.pub2
2. Chapman KR, Verbeek PR, White JG, et al. Effect of a Short Course of Prednisone in the Prevention of Early Relapse after the Emergency Room Treatment of Acute Asthma. New England Journal of Medicine 1991;324(12):788-94. doi: 10.1056/nejm199103213241202
3. UK A. Do no harm: Safer and better treatment options for people with asthma, 2021
4. Skov IR, Madsen H, Henriksen DP, et al. Low-dose oral corticosteroids in asthma associates with increased morbidity and mortality. Eur Respir J 2022;60(3) doi: 10.1183/13993003.03054-2021 [published Online First: 2022/02/12]

### Choice tasks

**Patients and HCPs were randomly assigned to completing 12 tasks from either block 1 or 2**

#### Block 1


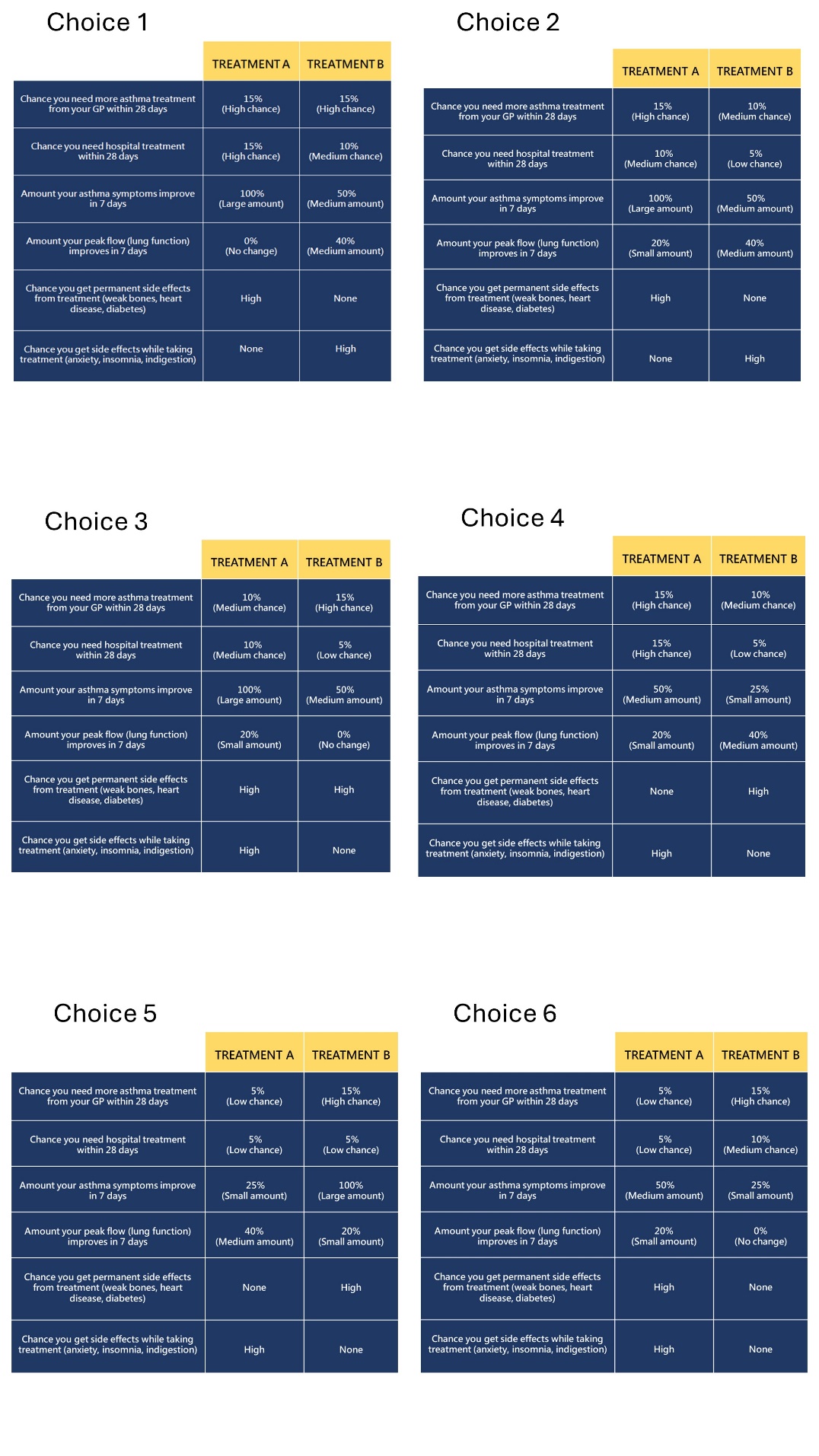


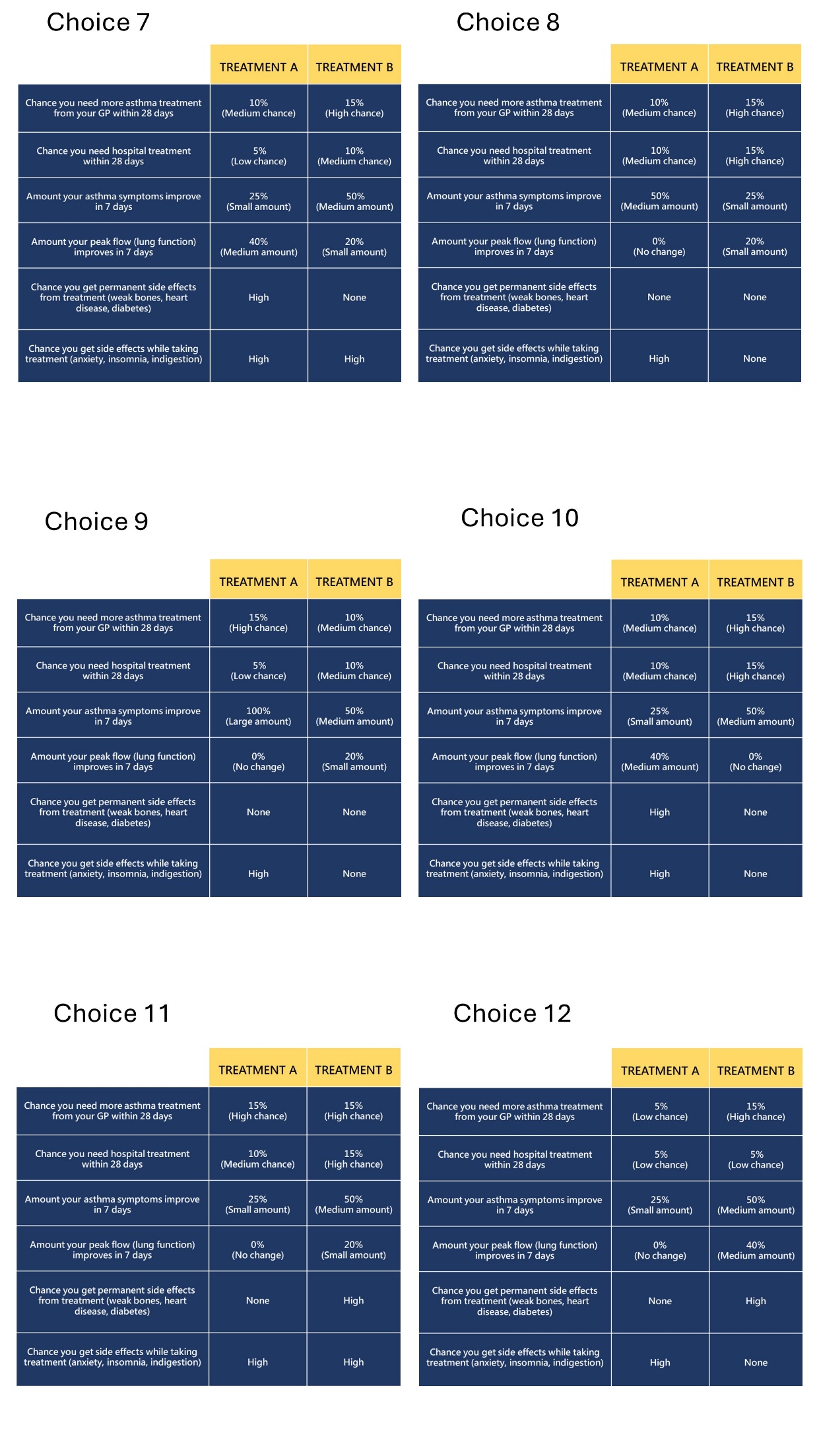


#### Block 2


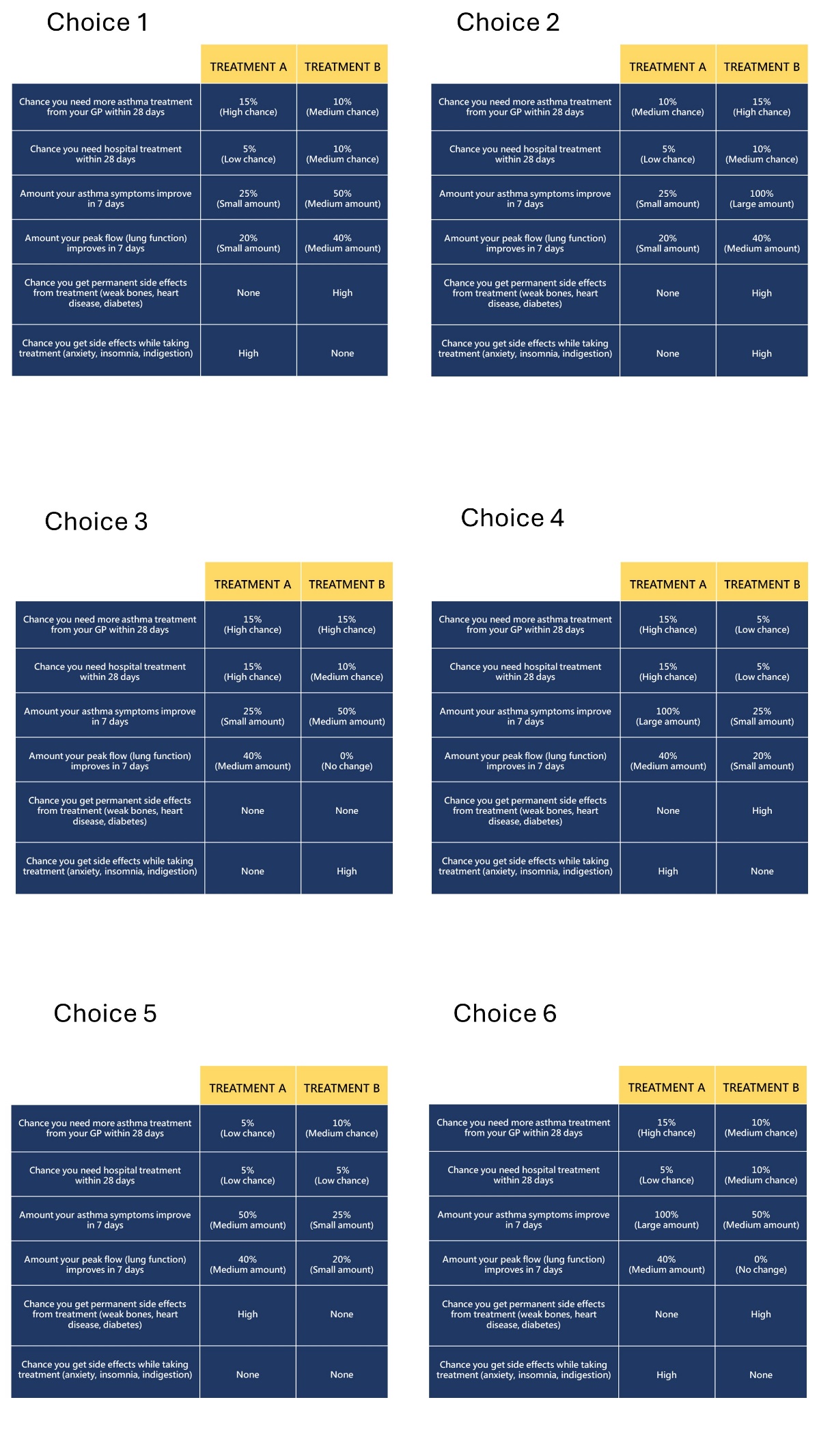


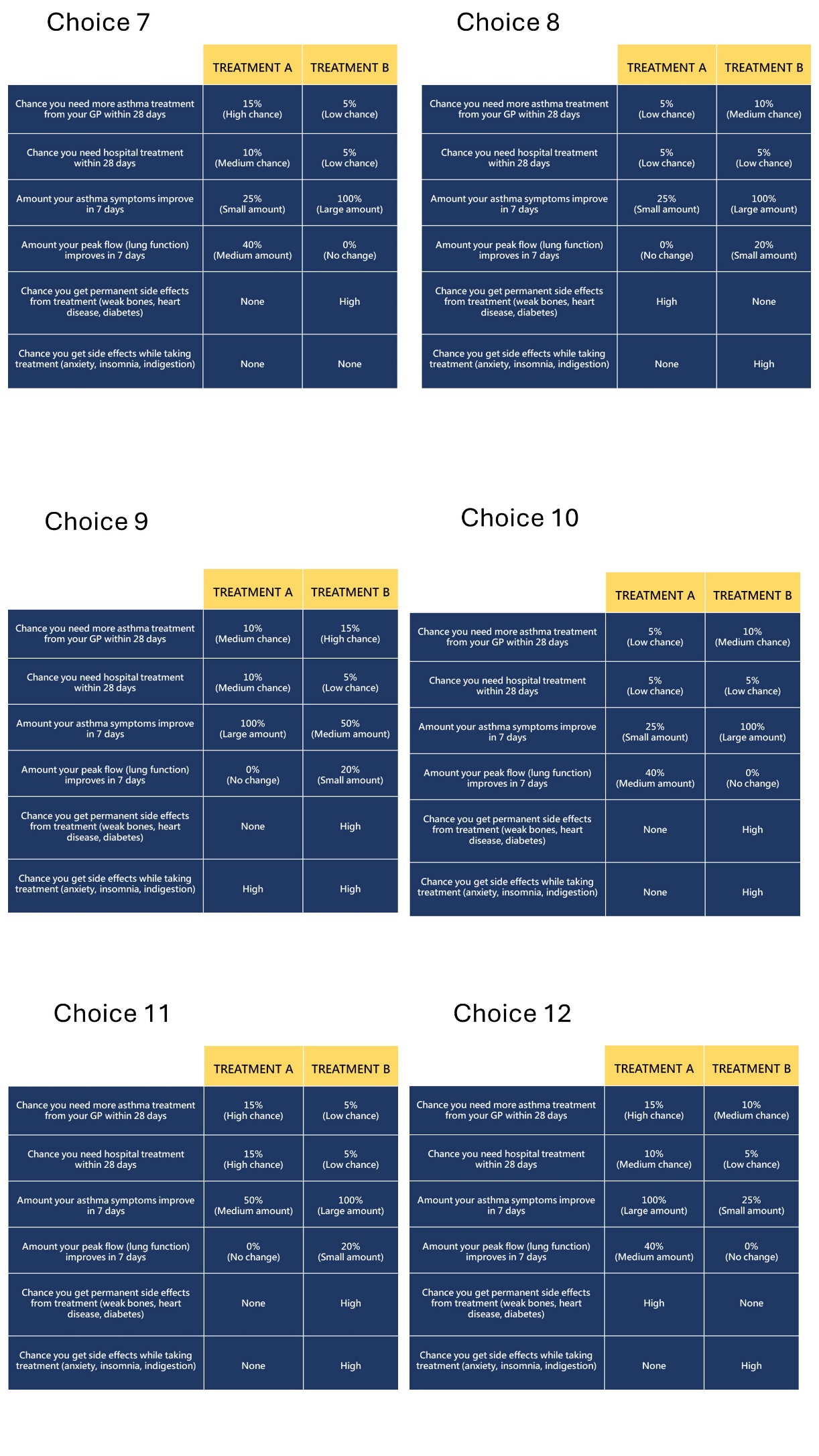


## Supplementary results

### Multinomial logit models

#### Robustness

The scale of the model between the Explanation 1 and Explanation 2 patient samples were not significantly different (robust t-ratio = -0.272).

The scale of the model between the UK and NZ patient samples were not significantly different (robust t-ratio = 0.843).

Hence, analysis of the pooled patient sample was appropriate.

The scale of the model between the UK and NZ HCP samples were also not significantly different (robust t-ratio = -0.589). Hence, analysis of the pooled HCP sample was appropriate.

There was a higher scale for the entire patient sample compared to the entire HCP sample (robust t-ratio 3.835). This implies that patients’ choices were more deterministic (less random) than the HCPs’ choices.

#### Sensitivity analysis

The mixed logit model of all patient responses showed similar results to the MNL model (supplementary table 8). There was a significant estimate for patients preferring the 5% hospitalisation level in the mixed logit model but not in the MNL model. However, the effects size for this level was small compared to other attribute levels.

The mixed logit of all HCP responses showed similar results to the MNL model (supplementary table 9).

Since the mixed logit sensitivity analysis supported the direction of estimates and their relative effect sizes found in the MNL models, we retained the more parsimonious MNL models as the primary analysis.

Supplementary table 1: MNL model of all patient responses

| **Attribute level** | **Estimate** | **Robust standard error** | **Robust t-ratio** |
| --- | --- | --- | --- |
| ASC Treatment A | -0.090 | 0.038 | -2.385 |
| ASC Treatment B | 0.000 | NA | NA |
| GP re-treatment 5% | 0.292 | 0.063 | 4.596 |
| GP re-treatment 10% | 0.212 | 0.032 | 6.649 |
| GP re-treatment 15% | 0.000 | NA | NA |
| Hospitalisation 5% | 0.092 | 0.061 | 1.507 |
| Hospitalisation 10% | 0.015 | 0.043 | 0.354 |
| Hospitalisation 15% | 0.000 | NA | NA |
| Symptom improvement 100% | 0.711 | 0.059 | 12.094 |
| Symptom improvement 50% | 0.241 | 0.037 | 6.577 |
| Symptom improvement 25% | 0.000 | NA | NA |
| Peak flow improvement 40% | 0.452 | 0.041 | 11.155 |
| Peak flow improvement 20% | 0.145 | 0.038 | 3.821 |
| Peak flow improvement 0% | 0.000 | NA | NA |
| Permanent SE none | 1.984 | 0.065 | 30.542 |
| Permanent SE high | 0.000 | NA | NA |
| Temporary SE none | 0.390 | 0.039 | 9.902 |
| Temporary SE high | 0.000 | NA | NA |
| Number of individuals  Number of observations  Number of estimated parameters  Log-likelihood of the fitted model | 824  9888  11  -4794.53 |  |  |

Supplementary table 2: MNL model of patients responses with socio-demographic interactions

| **Attribute level** | **Estimate** | **Robust standard error** | **Robust t-ratio** |
| --- | --- | --- | --- |
| ASC Treatment A | -0.092 | 0.038 | -2.406 |
| ASC Treatment B | 0.000 | NA | NA |
| GP re-treatment 5% | 0.312 | 0.126 | 2.467 |
| GP re-treatment 5% - Biologic | 0.009 | 0.138 | 0.064 |
| GP re-treatment 5% - ACT<15 | 0.169 | 0.126 | 1.341 |
| GP re-treatment 5% - Ever hospitalised | 0.029 | 0.121 | 0.243 |
| GP re-treatment 5% - Male | -0.054 | 0.128 | -0.424 |
| GP re-treatment 5% - Age over 65 | 0.189 | 0.124 | 1.527 |
| GP re-treatment 5% - Non-white | 0.020 | 0.186 | 0.106 |
| GP re-treatment 5% - No university education | -0.273 | 0.122 | -2.236 |
| GP re-treatment 10% | 0.235 | 0.066 | 3.569 |
| GP re-treatment 10% - Biologic | -0.035 | 0.078 | -0.455 |
| GP re-treatment 10% - ACT<15 | 0.026 | 0.070 | 0.364 |
| GP re-treatment 10% - Ever hospitalised | -0.001 | 0.066 | -0.016 |
| GP re-treatment 10% - Male | -0.124 | 0.067 | -1.853 |
| GP re-treatment 10% - Age over 65 | 0.096 | 0.071 | 1.359 |
| GP re-treatment 10% - Non-white | 0.007 | 0.099 | 0.075 |
| GP re-treatment 10% - No university education | -0.019 | 0.068 | -0.287 |
| GP re-treatment 15% | 0.000 | NA | NA |
| Hospitalisation 5% | 0.132 | 0.125 | 1.058 |
| Hospitalisation 5% - Biologic | 0.194 | 0.137 | 1.418 |
| Hospitalisation 5% - ACT<15 | 0.005 | 0.129 | 0.040 |
| Hospitalisation 5% - Ever hospitalised | -0.075 | 0.120 | -0.626 |
| Hospitalisation 5% - Male | -0.182 | 0.126 | -1.449 |
| Hospitalisation 5% - Age over 65 | 0.213 | 0.125 | 1.700 |
| Hospitalisation 5% - Non-white | -0.205 | 0.174 | -1.177 |
| Hospitalisation 5% - No university education | -0.041 | 0.122 | -0.336 |
| Hospitalisation 10% | 0.066 | 0.103 | 0.640 |
| Hospitalisation 10% - Biologic | 0.105 | 0.107 | 0.978 |
| Hospitalisation 10% - ACT<15 | 0.023 | 0.097 | 0.234 |
| Hospitalisation 10% - Ever hospitalised | -0.086 | 0.094 | -0.918 |
| Hospitalisation 10% - Male | -0.082 | 0.096 | -0.852 |
| Hospitalisation 10% - Age over 65 | 0.013 | 0.097 | 0.129 |
| Hospitalisation 10% - Non-white | -0.351 | 0.138 | -2.555 |
| Hospitalisation 10% - No university education | 0.081 | 0.093 | 0.872 |
| Hospitalisation 15% | 0.000 | NA | NA |
| Symptom improvement 100% | 0.809 | 0.126 | 6.418 |
| Symptom improvement 100% - Biologic | 0.130 | 0.140 | 0.928 |
| Symptom improvement 100% - ACT<15 | -0.037 | 0.129 | -0.288 |
| Symptom improvement 100% - Ever hospitalised | 0.119 | 0.123 | 0.965 |
| Symptom improvement 100% - Male | -0.106 | 0.129 | -0.823 |
| Symptom improvement 100% - Age over 65 | 0.260 | 0.130 | 2.004 |
| Symptom improvement 100% - Non-white | -0.058 | 0.173 | -0.334 |
| Symptom improvement 100% - No university education | -0.371 | 0.129 | -2.882 |
| Symptom improvement 50% | 0.281 | 0.080 | 3.507 |
| Symptom improvement 50% - Biologic | 0.087 | 0.092 | 0.952 |
| Symptom improvement 50% - ACT<15 | 0.036 | 0.083 | 0.433 |
| Symptom improvement 50% - Ever hospitalised | 0.077 | 0.076 | 1.006 |
| Symptom improvement 50% - Male | -0.107 | 0.083 | -1.283 |
| Symptom improvement 50% - Age over 65 | 0.248 | 0.084 | 2.957 |
| Symptom improvement 50% - Non-white | -0.025 | 0.107 | -0.235 |
| Symptom improvement 50% - No university education | -0.283 | 0.080 | -3.532 |
| Symptom improvement 25% | 0.000 | NA | NA |
| Peak flow improvement 40% | 0.530 | 0.085 | 6.202 |
| Peak flow improvement 40% - Biologic | -0.008 | 0.096 | -0.085 |
| Peak flow improvement 40% - ACT<15 | -0.212 | 0.091 | -2.333 |
| Peak flow improvement 40% - Ever hospitalised | -0.031 | 0.088 | -0.359 |
| Peak flow improvement 40% - Male | 0.084 | 0.088 | 0.950 |
| Peak flow improvement 40% - Age over 65 | 0.036 | 0.090 | 0.403 |
| Peak flow improvement 40% - Non-white | 0.012 | 0.138 | 0.085 |
| Peak flow improvement 40% - No university education | -0.015 | 0.092 | -0.168 |
| Peak flow improvement 20% | 0.107 | 0.078 | 1.367 |
| Peak flow improvement 20% - Biologic | 0.069 | 0.092 | 0.752 |
| Peak flow improvement 20% - ACT<15 | -0.084 | 0.081 | -1.044 |
| Peak flow improvement 20% - Ever hospitalised | 0.010 | 0.078 | 0.132 |
| Peak flow improvement 20% - Male | 0.116 | 0.082 | 1.406 |
| Peak flow improvement 20% - Age over 65 | -0.036 | 0.082 | -0.439 |
| Peak flow improvement 20% - Non-white | -0.075 | 0.106 | -0.705 |
| Peak flow improvement 20% - No university education | 0.058 | 0.079 | 0.740 |
| Peak flow improvement 0% | 0.000 | NA | NA |
| Permanent SE none | 2.723 | 0.149 | 18.303 |
| Permanent SE none - Biologic | -0.194 | 0.146 | -1.333 |
| Permanent SE none - ACT<15 | -0.402 | 0.132 | -3.045 |
| Permanent SE none - Ever hospitalised | -0.369 | 0.133 | -2.773 |
| Permanent SE none - Male | 0.273 | 0.136 | 2.005 |
| Permanent SE none - Age over 65 | -0.052 | 0.137 | -0.383 |
| Permanent SE none - Non-white | -0.314 | 0.178 | -1.769 |
| Permanent SE none - No university education | -0.739 | 0.136 | -5.428 |
| Permanent SE high | 0.000 | NA | NA |
| Temporary SE none | 0.398 | 0.086 | 4.634 |
| Temporary SE none - Biologic | -0.051 | 0.095 | -0.538 |
| Temporary SE none - ACT<15 | -0.066 | 0.089 | -0.745 |
| Temporary SE none - Ever hospitalised | -0.051 | 0.086 | -0.599 |
| Temporary SE none - Male | 0.040 | 0.088 | 0.451 |
| Temporary SE none - Age over 65 | 0.194 | 0.086 | 2.240 |
| Temporary SE none - Non-white | 0.104 | 0.143 | 0.731 |
| Temporary SE none - No university education | -0.043 | 0.087 | -0.492 |
| Temporary SE high | 0.000 | NA | NA |
| Number of individuals  Number of observations  Number of estimated parameters  Log-likelihood of the fitted model | 824  9888  81  -4617.53 |  |  |

Supplementary table 3: MNL model of poorly controlled asthma patients with a continuous treatment failure variable (for MRS calculation)

| **Attribute level** | **Estimate** | **Robust standard error** | **Robust t-ratio** |
| --- | --- | --- | --- |
| ASC Treatment A | -0.118 | 0.056 | -2.120 |
| ASC Treatment B | 0.000 | NA | NA |
| Treatment failure % (combined continuous GP re-treatment and hospitalisation) | -0.027 | 0.005 | -5.605 |
| Symptom improvement 100% | 0.680 | 0.098 | 6.961 |
| Symptom improvement 50% | 0.258 | 0.064 | 3.998 |
| Symptom improvement 25% | 0.000 | NA | NA |
| Peak flow improvement 40% | 0.320 | 0.067 | 4.781 |
| Peak flow improvement 20% | 0.098 | 0.061 | 1.598 |
| Peak flow improvement 0% | 0.000 | NA | NA |
| Permanent SE none | 1.565 | 0.104 | 14.986 |
| Permanent SE high | 0.000 | NA | NA |
| Temporary SE none | 0.342 | 0.066 | 5.168 |
| Temporary SE high | 0.000 | NA | NA |
| Number of individuals  Number of observations  Number of estimated parameters  Log-likelihood of the fitted model | 232  2784  8  -1554.8 |  |  |

Supplementary table 4: Marginal rate of substitution for treatment failure against permanent side effects in poorly controlled asthma patients

|  | **Estimate** | **Robust standard error** | **Robust t-ratio** |
| --- | --- | --- | --- |
| Marginal rate of substitution for treatment failure (%) in exchange for no permanent side effects | 57.8 | 10.106 | 5.720 |

Supplementary table 5: MNL model of patients randomised to Explanation 1 or Explanation 2

| **Attribute level** | **Estimate** | **Robust standard error** | **Robust t-ratio** |
| --- | --- | --- | --- |
| ASC Treatment A | -0.097 | 0.070 | -1.393 |
| ASC Treatment B | 0.000 | NA | NA |
| GP re-treatment 5% | 0.330 | 0.153 | 2.160 |
| GP re-treatment 5% - Explanation 2 | 0.088 | 0.202 | 0.437 |
| GP re-treatment 10% | 0.171 | 0.086 | 2.003 |
| GP re-treatment 10% - Explanation 2 | 0.162 | 0.118 | 1.364 |
| GP re-treatment 15% | 0.000 | NA | NA |
| Hospitalisation 5% | 0.143 | 0.147 | 0.971 |
| Hospitalisation 5% - Explanation 2 | -0.104 | 0.202 | -0.514 |
| Hospitalisation 10% | 0.061 | 0.107 | 0.567 |
| Hospitalisation 10% - Explanation 2 | -0.070 | 0.152 | -0.460 |
| Hospitalisation 15% | 0.000 | NA | NA |
| Symptom improvement 100% | 0.881 | 0.161 | 5.465 |
| Symptom improvement 100% - Explanation 2 | -0.112 | 0.226 | -0.496 |
| Symptom improvement 50% | 0.363 | 0.095 | 3.828 |
| Symptom improvement 50% - Explanation 2 | -0.147 | 0.134 | -1.102 |
| Symptom improvement 25% | 0.000 | NA | NA |
| Peak flow improvement 40% | 0.506 | 0.112 | 4.528 |
| Peak flow improvement 40% - Explanation 2 | -0.075 | 0.146 | -0.512 |
| Peak flow improvement 20% | 0.185 | 0.098 | 1.890 |
| Peak flow improvement 20% - Explanation 2 | -0.020 | 0.135 | -0.150 |
| Peak flow improvement 0% | 0.000 | NA | NA |
| Permanent SE none | 1.851 | 0.166 | 11.148 |
| Permanent SE none - Explanation 2 | -0.127 | 0.226 | -0.562 |
| Permanent SE high | 0.000 | NA | NA |
| Temporary SE none | 0.396 | 0.097 | 4.084 |
| Temporary SE none - Explanation 2 | -0.154 | 0.138 | -1.118 |
| Temporary SE high | 0.000 | NA | NA |
| Number of individuals  Number of observations  Number of estimated parameters  Log-likelihood of the fitted model | 222  2664  21  -1392.18 |  |  |

Supplementary table 6: MNL model of all HCP responses

| **Attribute level** | **Estimate** | **Robust standard error** | **Robust t-ratio** |
| --- | --- | --- | --- |
| ASC Treatment A | -0.115 | 0.072 | -1.600 |
| ASC Treatment B | 0.000 | NA | NA |
| GP re-treatment 5% | 0.737 | 0.126 | 5.853 |
| GP re-treatment 10% | 0.264 | 0.070 | 3.794 |
| GP re-treatment 15% | 0.000 | NA | NA |
| Hospitalisation 5% | 0.623 | 0.136 | 4.584 |
| Hospitalisation 10% | 0.305 | 0.090 | 3.393 |
| Hospitalisation 15% | 0.000 | NA | NA |
| Symptom improvement 100% | 1.245 | 0.110 | 11.270 |
| Symptom improvement 50% | 0.411 | 0.067 | 6.146 |
| Symptom improvement 25% | 0.000 | NA | NA |
| Peak flow improvement 40% | 0.340 | 0.088 | 3.878 |
| Peak flow improvement 20% | 0.023 | 0.079 | 0.296 |
| Peak flow improvement 0% | 0.000 | NA | NA |
| Permanent SE none | 1.757 | 0.108 | 16.269 |
| Permanent SE high | 0.000 | NA | NA |
| Temporary SE none | 0.511 | 0.075 | 6.843 |
| Temporary SE high | 0.000 | NA | NA |
| Number of individuals  Number of observations  Number of estimated parameters  Log-likelihood of the fitted model | 171  2052  11  -1140.97 |  |  |

Supplementary table 7: MNL model of all HCP responses with socio-demographic interactions

| **Attribute level** | **Estimate** | **Robust standard error** | **Robust t-ratio** |
| --- | --- | --- | --- |
| ASC Treatment A | -0.130 | 0.073 | -1.789 |
| ASC Treatment B | 0.000 | NA | NA |
| GP re-treatment 5% | 0.818 | 0.239 | 3.425 |
| GP re-treatment 5% - Primary care setting | -0.072 | 0.286 | -0.252 |
| GP re-treatment 5% - Male | -0.130 | 0.252 | -0.516 |
| GP re-treatment 5% - Non-doctor HCP | -0.206 | 0.287 | -0.718 |
| GP re-treatment 5% - Age over 40 | 0.142 | 0.242 | 0.586 |
| GP re-treatment 10% | 0.599 | 0.157 | 3.812 |
| GP re-treatment 10% - Primary care setting | 0.055 | 0.155 | 0.353 |
| GP re-treatment 10% - Male | -0.343 | 0.156 | -2.193 |
| GP re-treatment 10% - Non-doctor HCP | -0.127 | 0.170 | -0.749 |
| GP re-treatment 10% - Age over 40 | -0.355 | 0.139 | -2.550 |
| GP re-treatment 15% | 0.000 | NA | NA |
| Hospitalisation 5% | -0.032 | 0.248 | -0.130 |
| Hospitalisation 5% - Primary care setting | 0.004 | 0.286 | 0.014 |
| Hospitalisation 5% - Male | 0.924 | 0.275 | 3.360 |
| Hospitalisation 5% - Non-doctor HCP | 0.288 | 0.254 | 1.137 |
| Hospitalisation 5% - Age over 40 | 0.441 | 0.250 | 1.760 |
| Hospitalisation 10% | -0.045 | 0.180 | -0.249 |
| Hospitalisation 10% - Primary care setting | -0.043 | 0.200 | -0.213 |
| Hospitalisation 10% - Male | 0.565 | 0.215 | 2.630 |
| Hospitalisation 10% - Non-doctor HCP | 0.198 | 0.207 | 0.956 |
| Hospitalisation 10% - Age over 40 | 0.200 | 0.180 | 1.110 |
| Hospitalisation 15% | 0.000 | NA | NA |
| Symptom improvement 100% | 1.137 | 0.215 | 5.289 |
| Symptom improvement 100% - Primary care setting | 0.045 | 0.294 | 0.154 |
| Symptom improvement 100% - Male | 0.331 | 0.232 | 1.426 |
| Symptom improvement 100% - Non-doctor HCP | 0.247 | 0.321 | 0.767 |
| Symptom improvement 100% - Age over 40 | -0.089 | 0.237 | -0.375 |
| Symptom improvement 50% | 0.035 | 0.118 | 0.294 |
| Symptom improvement 50% - Primary care setting | 0.045 | 0.184 | 0.246 |
| Symptom improvement 50% - Male | 0.478 | 0.140 | 3.415 |
| Symptom improvement 50% - Non-doctor HCP | 0.442 | 0.217 | 2.042 |
| Symptom improvement 50% - Age over 40 | 0.170 | 0.144 | 1.175 |
| Symptom improvement 25% | 0.000 | NA | NA |
| Peak flow improvement 40% | 0.223 | 0.178 | 1.250 |
| Peak flow improvement 40% - Primary care setting | 0.040 | 0.193 | 0.205 |
| Peak flow improvement 40% - Male | 0.102 | 0.203 | 0.505 |
| Peak flow improvement 40% - Non-doctor HCP | 0.445 | 0.229 | 1.942 |
| Peak flow improvement 40% - Age over 40 | -0.065 | 0.194 | -0.334 |
| Peak flow improvement 20% | -0.074 | 0.148 | -0.503 |
| Peak flow improvement 20% - Primary care setting | 0.082 | 0.179 | 0.456 |
| Peak flow improvement 20% - Male | 0.300 | 0.192 | 1.557 |
| Peak flow improvement 20% - Non-doctor HCP | 0.271 | 0.210 | 1.288 |
| Peak flow improvement 20% - Age over 40 | -0.204 | 0.165 | -1.236 |
| Peak flow improvement 0% | 0.000 | NA | NA |
| Permanent SE none | 1.603 | 0.237 | 6.768 |
| Permanent SE none - Primary care setting | 0.061 | 0.268 | 0.229 |
| Permanent SE none - Male | 0.404 | 0.234 | 1.725 |
| Permanent SE none - Non-doctor HCP | 0.432 | 0.297 | 1.453 |
| Permanent SE none - Age over 40 | -0.148 | 0.227 | -0.651 |
| Permanent SE high | 0.000 | NA | NA |
| Temporary SE none | 0.417 | 0.170 | 2.446 |
| Temporary SE none - Primary care setting | 0.081 | 0.192 | 0.425 |
| Temporary SE none - Male | 0.136 | 0.169 | 0.806 |
| Temporary SE none - Non-doctor HCP | 0.218 | 0.176 | 1.239 |
| Temporary SE none - Age over 40 | -0.048 | 0.163 | -0.297 |
| Temporary SE high | 0.000 | NA | NA |
| Number of individuals  Number of observations  Number of estimated parameters  Log-likelihood of the fitted model | 171  2052  51  -1112.86 |  |  |

Supplementary table 8: MNL model of HCPs with a continuous treatment failure variable (for MRS calculation)

| **Attribute level** | **Estimate** | **Robust standard error** | **Robust t-ratio** |
| --- | --- | --- | --- |
| ASC Treatment A | -0.087 | 0.058 | -1.503 |
| ASC Treatment B | 0.000 | NA | NA |
| Treatment failure (combined continuous GP re-treatment and hospitalisation) | -0.067 | 0.007 | -9.242 |
| Symptom improvement 100% | 1.259 | 0.116 | 10.820 |
| Symptom improvement 50% | 0.431 | 0.069 | 6.218 |
| Symptom improvement 25% | 0.000 | NA | NA |
| Peak flow improvement 40% | 0.338 | 0.087 | 3.894 |
| Peak flow improvement 20% | 0.035 | 0.078 | 0.447 |
| Peak flow improvement 0% | 0.000 | NA | NA |
| Permanent SE none | 1.752 | 0.107 | 16.379 |
| Permanent SE high | 0.000 | NA | NA |
| Temporary SE none | 0.521 | 0.074 | 7.057 |
| Temporary SE high | 0.000 | NA | NA |
| Number of individuals  Number of observations  Number of estimated parameters  Log-likelihood of the fitted model | 171  2052  8  -1141.34 |  |  |

Supplementary table 9: Marginal rate of substitution for treatment failure against permanent side effects in HCPs

|  | **Estimate** | **Robust standard error** | **Robust t-ratio** |
| --- | --- | --- | --- |
| Marginal rate of substitution for treatment failure (%) in exchange for no permanent side effects | 26.3 | 2.844 | 9.230 |

**Mixed logit models**

Supplementary table 10: Mixed logit model of all patient responses

The mean and the standard deviation (SD) of the normal distribution were estimated for each parameter.

| **Attribute level** | **Estimate** | **Robust standard error** | **Robust t-ratio** |
| --- | --- | --- | --- |
| ASC Treatment A | -0.156 | 0.053 | -2.956 |
| ASC Treatment B | 0.000 | NA | NA |
| Mean GP re-treatment 5% | 0.482 | 0.089 | 5.406 |
| SD GP re-treatment 5% | 0.511 | 0.143 | 3.579 |
| Mean GP re-treatment 10% | 0.315 | 0.048 | 6.512 |
| SD GP re-treatment 10% | 0.043 | 0.044 | 0.971 |
| Mean GP re-treatment 15% | 0.000 | NA | NA |
| SD GP re-treatment 15% | 0.000 | NA | NA |
| Mean Hospitalisation 5% | 0.220 | 0.094 | 2.343 |
| SD Hospitalisation 5% | 0.268 | 0.150 | 1.784 |
| Mean Hospitalisation 10% | 0.066 | 0.068 | 0.971 |
| SD Hospitalisation 10% | 0.003 | 0.029 | 0.101 |
| Mean Hospitalisation 15% | 0.000 | NA | NA |
| SD Hospitalisation 15% | 0.000 | NA | NA |
| Mean Symptom improvement 100% | 1.194 | 0.101 | 11.788 |
| SD Symptom improvement 100% | 0.813 | 0.095 | 8.558 |
| Mean Symptom improvement 50% | 0.458 | 0.061 | 7.553 |
| SD Symptom improvement 50% | 0.016 | 0.114 | 0.144 |
| Mean Symptom improvement 25% | 0.000 | NA | NA |
| SD Symptom improvement 25% | 0.000 | NA | NA |
| Mean Peak flow improvement 40% | 0.746 | 0.067 | 11.118 |
| SD Peak flow improvement 40% | 0.585 | 0.086 | 6.820 |
| Mean Peak flow improvement 20% | 0.202 | 0.055 | 3.688 |
| SD Peak flow improvement 20% | 0.397 | 0.108 | 3.695 |
| Mean Peak flow improvement 0% | 0.000 | NA | NA |
| SD Peak flow improvement 0% | 0.000 | NA | NA |
| Mean Permanent SE none | 3.652 | 0.158 | 23.053 |
| SD Permanent SE none | 2.263 | 0.100 | 22.713 |
| Mean Permanent SE high | 0.000 | NA | NA |
| SD Permanent SE high | 0.000 | NA | NA |
| Mean Temporary SE none | 0.664 | 0.066 | 10.064 |
| SD Temporary SE none | 0.870 | 0.066 | 13.204 |
| Mean Temporary SE high | 0.000 | NA | NA |
| SD Temporary SE high | 0.000 | NA | NA |
| Number of individuals  Number of observations  Number of estimated parameters  Number of inter-individual draws (MLHS)  Log-likelihood of the fitted model | 824  9888  21  2000  -4146.64 |  |  |

Supplementary table 11: Mixed logit model of all HCP responses

The mean and the standard deviation (SD) of the normal distribution were estimated for each parameter.

| **Attribute level** | **Estimate** | **Robust standard error** | **Robust t-ratio** |
| --- | --- | --- | --- |
| ASC Treatment A | -0.232 | 0.113 | -2.056 |
| ASC Treatment B | 0.000 | NA | NA |
| Mean GP re-treatment 5% | 1.357 | 0.234 | 5.805 |
| SD GP re-treatment 5% | 1.231 | 0.236 | 5.212 |
| Mean GP re-treatment 10% | 0.469 | 0.118 | 3.990 |
| SD GP re-treatment 10% | 0.309 | 0.348 | 0.888 |
| Mean GP re-treatment 15% | 0.000 | NA | NA |
| SD GP re-treatment 15% | 0.000 | NA | NA |
| Mean Hospitalisation 5% | 0.940 | 0.230 | 4.085 |
| SD Hospitalisation 5% | 1.016 | 0.211 | 4.818 |
| Mean Hospitalisation 10% | 0.412 | 0.148 | 2.783 |
| SD Hospitalisation 10% | 0.062 | 0.056 | 1.122 |
| Mean Hospitalisation 15% | 0.000 | NA | NA |
| SD Hospitalisation 15% | 0.000 | NA | NA |
| Mean Symptom improvement 100% | 2.135 | 0.238 | 8.970 |
| SD Symptom improvement 100% | 0.802 | 0.209 | 3.835 |
| Mean Symptom improvement 50% | 0.708 | 0.128 | 5.513 |
| SD Symptom improvement 50% | 0.042 | 0.120 | 0.351 |
| Mean Symptom improvement 25% | 0.000 | NA | NA |
| SD Symptom improvement 25% | 0.000 | NA | NA |
| Mean Peak flow improvement 40% | 0.618 | 0.159 | 3.874 |
| SD Peak flow improvement 40% | 1.021 | 0.169 | 6.028 |
| Mean Peak flow improvement 20% | 0.061 | 0.132 | 0.465 |
| SD Peak flow improvement 20% | 0.680 | 0.162 | 4.193 |
| Mean Peak flow improvement 0% | 0.000 | NA | NA |
| SD Peak flow improvement 0% | 0.000 | NA | NA |
| Mean Permanent SE none | 3.133 | 0.296 | 10.598 |
| SD Permanent SE none | 1.819 | 0.206 | 8.844 |
| Mean Permanent SE high | 0.000 | NA | NA |
| SD Permanent SE high | 0.000 | NA | NA |
| Mean Temporary SE none | 0.878 | 0.144 | 6.111 |
| SD Temporary SE none | 0.725 | 0.152 | 4.775 |
| Mean Temporary SE high | 0.000 | NA | NA |
| SD Temporary SE high | 0.000 | NA | NA |
| Number of individuals  Number of observations  Number of estimated parameters  Number of inter-individual draws (MLHS)  Log-likelihood of the fitted model | 171  2052  21  2000  -1029.84 |  |  |

Supplementary table 12: MNL model of all patient responses re-weighted for patient educational attainment

| **Attribute level** | **Estimate** | **Robust standard error** | **Robust t-ratio** |
| --- | --- | --- | --- |
| ASC Treatment A | -0.093 | 0.030 | -2.193 |
| ASC Treatment B | 0.000 | NA | NA |
| GP re-treatment 5% | 0.245 | 0.052 | 3.463 |
| GP re-treatment 10% | 0.209 | 0.036 | 5.873 |
| GP re-treatment 15% | 0.000 | NA | NA |
| Hospitalisation 5% | 0.091 | 0.063 | 1.288 |
| Hospitalisation 10% | 0.041 | 0.051 | 0.846 |
| Hospitalisation 15% | 0.000 | NA | NA |
| Symptom improvement 100% | 0.638 | 0.054 | 9.785 |
| Symptom improvement 50% | 0.185 | 0.040 | 4.598 |
| Symptom improvement 25% | 0.000 | NA | NA |
| Peak flow improvement 40% | 0.442 | 0.037 | 9.733 |
| Peak flow improvement 20% | 0.158 | 0.036 | 3.823 |
| Peak flow improvement 0% | 0.000 | NA | NA |
| Permanent SE none | 1.783 | 0.043 | 25.504 |
| Permanent SE high | 0.000 | NA | NA |
| Temporary SE none | 0.380 | 0.038 | 8.403 |
| Temporary SE high | 0.000 | NA | NA |
| Number of individuals  Number of observations  Number of estimated parameters  Log-likelihood of the fitted model | 824  9888  11  -5088 |  |  |

Supplementary Figure 1 – Forecasted choices of OCS versus placebo by patients and HCPs

This bar chart compares the predicted choice probability that patients and HCPs would choose OCS treatment versus a placebo-equivalent treatment. Probabilities use the model estimates and specific attribute values to forecast for each treatment. For OCS: 5% relapse to GP, 5% relapse to hospital, 100% symptom improvement, 40% peak flow improvement, and high risk of permanent and temporary side effects. For placebo: 15% relapse to GP, 15% relapse to hospital, 25% symptom improvement, 0% peak flow improvement, and no permanent or temporary side effects.

**
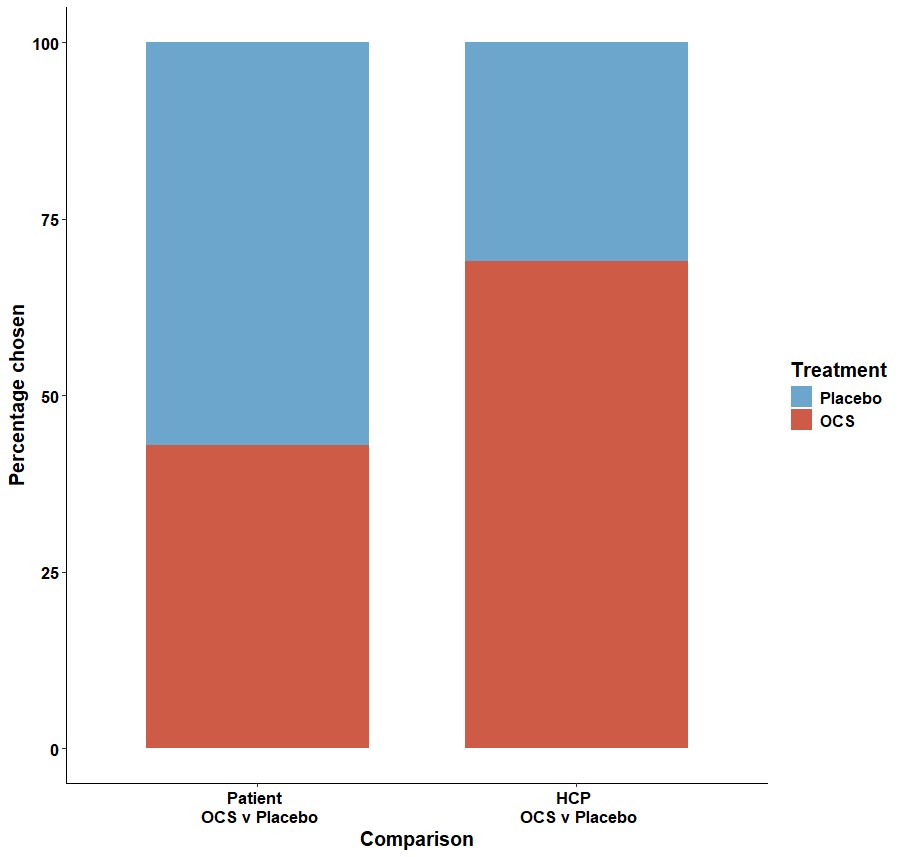
**

Supplementary Figure 2 – The interaction of socio-demographic characteristics on healthcare professional preferences

Forest plot showing the influence of socio-demographic factors on healthcare professional preferences for different levels of risks and benefits. An OR >1 denotes stronger preference and an OR <1 denotes weaker preference.

**
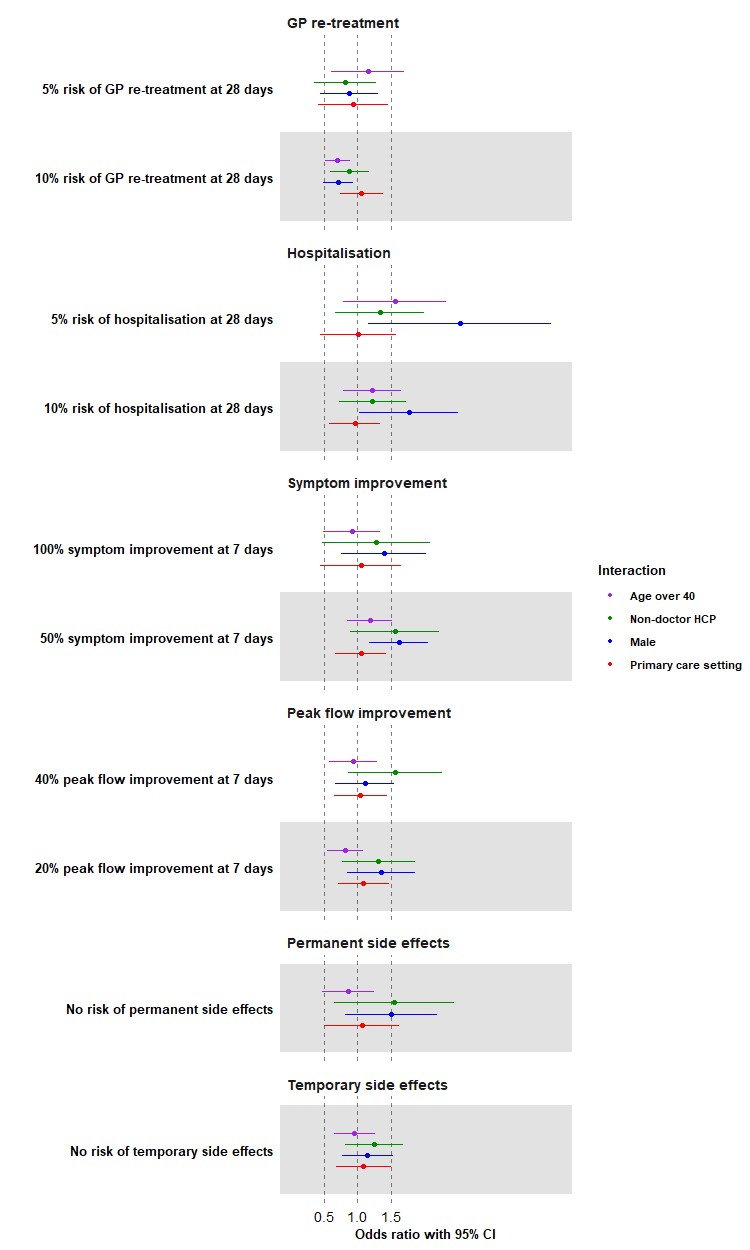
**

**References:**

1. Hensher DA, Rose JM, Greene WH. Applied Choice Analysis. 2 ed. Cambridge: Cambridge University Press 2015.

2. Mott DJ, Chami N, Tervonen T. Reporting Quality of Marginal Rates of Substitution in Discrete Choice Experiments That Elicit Patient Preferences. *Value Health* 2020;23(8):979-84. doi: 10.1016/j.jval.2020.04.1831 [published Online First: 2020/08/24]

3. Cook JA, Julious SA, Sones W, et al. Practical help for specifying the target difference in sample size calculations for RCTs: the DELTA(2) five-stage study, including a workshop. *Health Technol Assess* 2019;23(60):1-88. doi: 10.3310/hta23600 [published Online First: 2019/10/30]

4. Rowe BH, Spooner C, Ducharme F, et al. Corticosteroids for preventing relapse following acute exacerbations of asthma. *Cochrane Database of Systematic Reviews* 2007(3) doi: 10.1002/14651858.CD000195.pub2

5. Ramakrishnan S, Jeffers H, Langford-Wiley B, et al. Point of care blood eosinophil guided oral prednisolone for COPD exacerbations: a multi-centre double blind randomised controlled trial (The STARR2 trial). *European Respiratory Journal* 2022;60(suppl 66):4728. doi: 10.1183/13993003.congress-2022.4728

6. Howell I, Howell A, Ramakrishnan S, et al. How have we measured trial outcomes of asthma attack treatment? A systematic review. *ERJ Open Res* 2024;10(1) doi: 10.1183/23120541.00660-2023 [published Online First: 2024/02/27]

7. Bonini M, Di Paolo M, Bagnasco D, et al. Minimal clinically important difference for asthma endpoints: an expert consensus report. *Eur Respir Rev* 2020;29(156) doi: 10.1183/16000617.0137-2019 [published Online First: 2020/06/06]

8. Swait J, Louviere J. The Role of the Scale Parameter in the Estimation and Comparison of Multinomial Logit Models. *Journal of Marketing Research* 1993;30(3):305-14. doi: 10.2307/3172883

9. Vass CM, Wright S, Burton M, et al. Scale Heterogeneity in Healthcare Discrete Choice Experiments: A Primer. *Patient* 2018;11(2):167-73. doi: 10.1007/s40271-017-0282-4 [published Online First: 2017/10/17]

10. Buckell J, Hess S. Stubbing out hypothetical bias: improving tobacco market predictions by combining stated and revealed preference data. *J Health Econ* 2019;65:93-102. doi: 10.1016/j.jhealeco.2019.03.011 [published Online First: 2019/04/16]

11. McDowell PJ, Diver S, Yang F, et al. The inflammatory profile of exacerbations in patients with severe refractory eosinophilic asthma receiving mepolizumab (the MEX study): a prospective observational study. *Lancet Respir Med* 2021 doi: 10.1016/s2213-2600(21)00004-7 [published Online First: 2021/05/11]

12. McDowell PJ, Busby J, Hanratty CE, et al. Exacerbation Profile and Risk Factors in a Type-2-Low Enriched Severe Asthma Cohort: A Clinical Trial to Assess Asthma Exacerbation Phenotypes. *Am J Respir Crit Care Med* 2022;206(5):545-53. doi: 10.1164/rccm.202201-0129OC [published Online First: 2022/05/14]

13. Hensher DA, Greene WH. The Mixed Logit model: The state of practice. *Transportation* 2003;30(2):133-76. doi: 10.1023/A:1022558715350

14. Train KE. Discrete Choice Methods with Simulation. Cambridge: Cambridge University Press 2003.

15. Hess S, Train KE, Polak JW. On the use of a Modified Latin Hypercube Sampling (MLHS) method in the estimation of a Mixed Logit Model for vehicle choice. *Transportation Research Part B: Methodological* 2006;40(2):147-63. doi: <https://doi.org/10.1016/j.trb.2004.10.005>
